# Ionotropic Receptors as a Driving Force behind Human Synapse Establishment

**DOI:** 10.1093/molbev/msaa252

**Published:** 2020-09-28

**Authors:** Lucas Henriques Viscardi, Danilo Oliveira Imparato, Maria Cátira Bortolini, Rodrigo Juliani Siqueira Dalmolin

**Affiliations:** 1 Departamento de Genética, Instituto de Biociências, Universidade Federal do Rio Grande do Sul, Porto Alegre, RS, Brazil; 2 Bioinformatics Multidisciplinary Environment—BioME, IMD, Federal University of Rio Grande do Norte, Natal, RN, Brazil; 3 Department of Biochemistry, CB, Federal University of Rio Grande do Norte, Natal, RN, Brazil

**Keywords:** neurotransmission, synapse evolution, systems biology, synapse network, ionotropic receptor

## Abstract

The origin of nervous systems is a main theme in biology and its mechanisms are largely underlied by synaptic neurotransmission. One problem to explain synapse establishment is that synaptic orthologs are present in multiple aneural organisms. We questioned how the interactions among these elements evolved and to what extent it relates to our understanding of the nervous systems complexity. We identified the human neurotransmission gene network based on genes present in GABAergic, glutamatergic, serotonergic, dopaminergic, and cholinergic systems. The network comprises 321 human genes, 83 of which act exclusively in the nervous system. We reconstructed the evolutionary scenario of synapse emergence by looking for synaptic orthologs in 476 eukaryotes. The Human–Cnidaria common ancestor displayed a massive emergence of neuroexclusive genes, mainly ionotropic receptors, which might have been crucial to the evolution of synapses. Very few synaptic genes had their origin after the Human–Cnidaria common ancestor. We also identified a higher abundance of synaptic proteins in vertebrates, which suggests an increase in the synaptic network complexity of those organisms.

## Introduction

The high complexity of human cognition is associated with the central nervous system and its synaptic organization (Silbereis et al. 2016). At the molecular level, however, the synapse is the most fundamental unity of neural communication. The synapse relies on several molecules such as neurotransmitters, membrane receptors, ion channels, and signal transduction proteins, acting together in a complex molecular interaction network. Their orthologs have been described in evolutionarily distant taxa, including species without neurons (Sakarya et al. 2007; Burkhardt et al. 2011; Emes and Grant 2011, 2012; Suga et al. 2013; Burkhardt 2015). Therefore, gene products involved in synapses evolved in different scenarios, from signal transduction in prokaryotes to synapses establishment itself (Bocquet et al. 2007; Emes and Grant 2011; Bertozzi et al. 2016). Additionally, most of them also work in other signaling processes in modern organisms (Emes and Grant 2012). Despite the efforts to identify broadly expressed neuronal genes, there are no genes ubiquitously distributed across neural animals exclusively related to nerve cells (Moroz and Kohn 2015). Given the complexity of the synaptic network, the relationships among its synaptic elements could better explain the advent of neurotransmission.

The main synaptic systems in humans include the neurotransmitters γ-aminobutyric acid (GABA), glutamate, serotonin, dopamine, and acetylcholine (Kondziella 2017; Wideman et al. 2018). GABA mainly exerts inhibitory function, whereas glutamate predominantly works as an excitatory neurotransmitter. Serotonin, dopamine, and acetylcholine can be either excitatory or inhibitory, depending on the tissue and the receptor they interact with (Belousov et al. 2001; Rho and Storey 2001). Neurotransmitter receptors can be divided into two major groups, considering their physiology and molecular structure: ionotropic receptors and metabotropic receptors. Ionotropic receptors directly gate ion flow into cells, leading to either excitatory or inhibitory responses in postsynaptic neurons. On the other hand, metabotropic receptors indirectly modulate the ion flow by activating guanine nucleotide-binding proteins (G proteins), triggering intracellular cascades that might ultimately induce excitatory or inhibitory responses (Leys 2015).

It has long been thought that cnidarians were the first organisms to evolve a nervous system in the form of diffuse nerve nets (Krishnan and Schioth 2015). However, an increasing body of evidence places ctenophores as a sister group to all other metazoans, suggesting that nerve cells had independent origins in cnidarians and ctenophores (Norekian and Moroz 2020). It has also been proposed that synaptic communication was present in the animals common ancestor and was posteriorly lost in Porifera and Placozoa (Moroz et al. 2014; Moroz 2015; Pisani et al. 2015). Regardless of its origin, it is well understood that the human synaptic machinery was vertically inherited from the last common ancestor (LCA) of mammals and cnidarians (Emes and Grant 2012). Therefore, understanding the steps of human neurotransmission gene network evolution might help to understand the establishment of the synapse itself.

Our goal here was to reconstruct the evolutionary scenario of the human neurotransmission gene network establishment. We inferred that the network based on genes present in the five major neurotransmitter systems: GABAergic, glutamatergic, serotonergic, dopaminergic, and cholinergic. We identified network nodes exclusively related to nervous tissues based on gene annotation and gene expression. We also looked for the earliest vertically inherited genetic archetype of each network node (Castro et al. 2008) to trace back the evolutionary origin of the human neurotransmission gene network. According to our results, the Human–Cnidaria LCA is marked by the emergence of several receptor families. Ionotropic receptors represent the majority of the receptors rooted at the Human–Cnidaria LCA, whereas the majority of metabotropic receptors are rooted before the establishment of synapses. Our results suggest that, regardless of the presence of synapses in Ctenophores, the expansion of neurotransmitter receptors in a Human–Cnidaria LCA, especially ionotropic receptors, had a critical role in the evolution of synapses.

## Results

### Human Neurotransmission Gene Network

**Fig. 1.**
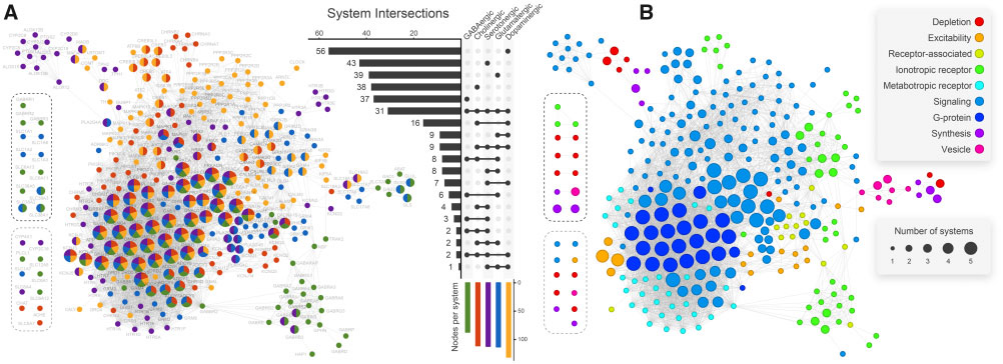
Human neurotransmission PPI network. Nodes represent proteins, whereas edges represent the interactions among them. Nodes enclosed by dashed lines either did not connect to the largest connected component (black dashed lines) or did not connect at all (gray dashed lines). Node size is proportional to the number of neuronal pathways it participates (i.e., GABAergic, cholinergic, serotonergic, glutamatergic, and dopaminergic). Node color represents the pathway(s) a node belongs to (*A*) or the node corresponding function in neurotransmission (*B*). The central UpSet diagram depicts how nodes are distributed among pathways (e.g., 31 genes take part in all five pathways). Colored bars at the bottom show absolute node count on each pathway (e.g., the dopaminergic pathway comprises 131 genes, 56 of which take part in no other pathways). Linked dots indicate node overlap among pathways, whereas horizontal bars indicate overlap sizes.

Our analysis started by selecting human genes involved in synaptic neurotransmission. We identified 325 genes taking part in the five major neurotransmission pathways (dopaminergic, glutamatergic, serotonergic, cholinergic, and GABAergic; supplementary table S1, Supplementary Material online). Three hundred and twenty-one of these genes were present in the STRING database and correspond to neurotransmission nodes (NNs). Figure 1 depicts the obtained protein–protein interaction (PPI) network, which we define as the human neurotransmission gene network. It comprises 297 NNs in the largest connected component, 13 NNs in small isolated components, and 11 unconnected NNs. Roughly a third of NNs take part in multiple synaptic pathways. NNs placed in the network core are prone to be associated with more than one system, and about 10% of them (31 NNs) pervade all five systems (fig. 1). The dopaminergic system is the largest one, comprising 131 NNs, among which 56 (42%) are uniquely dopaminergic related (fig. 1*A*). By contrast, the GABAergic system is the smallest one, comprising 89 NNs, 37 (41%) of them being uniquely GABAergic related. Cholinergic, serotonergic, and glutamatergic systems have a very similar number of nodes (112, 115, and 114, respectively). Dopaminergic-related nodes are found throughout the network. GABAergic-exclusive nodes are mainly found at the network periphery, and roughly 30% of them are not present in the largest connected component.

According to figure 1, all five systems are intimately interconnected. An example is the solute carrier family 18 (*SLC18*) and solute carrier family 17 (*SLC17*), which involves NNs of the five neurotransmitter systems (fig. 1). Glutamate activity was already suggested to play a significant role in dopamine release in the ventral tegmental area (Fitzgerald et al. 2012). The vesicular glutamate transporter *SLC17A6*, a mediator of the glutamate uptake into synaptic vesicles at presynaptic nerve terminals of excitatory neural cells, mediates an increase in dopamine vesicle content in mouse midbrain neurons. This transporter colocalized with *SLC18A2* (a monoamine transporter) in mammalian dopaminergic neurons, a mechanism conserved in mammals and also identified in *Drosophila melanogaster*. This pathway is crucial to tune the amount of neurotransmitter released per vesicle in response to the demands of repeated neuronal firing (Aguilar et al. 2017). From a physiological perspective, such interactions among the systems can also be observed. As an example, dopamine can modulate GABA release onto cholinergic interneurons in the striatum (Momiyama and Koga 2001; Kosillo et al. 2016) and kainate receptors from the *GRIK* family can also modulate GABA transmission (Rodríguez-Moreno and Lerma 1998; Negrete-Díaz et al. 2018). Additionally, some ionotropic glutamate receptor (iGluR) subunits are also known to regulate extracellular dopamine concentration in the striatum, therefore taking part in both glutamatergic and dopaminergic pathways (Borland and Michael 2004).

The NNs were also classified according to their function in neurotransmission: neurotransmitter depletion (clearance from synaptic cleft and degradation), cellular excitability, ionotropic receptors (ligand-gated ion channels), metabotropic receptors (G protein-coupled receptors), receptor associated, intracellular signaling, G proteins, neurotransmitter synthesis (including precursor transport), and vesicle related (fig. 1*B*). It can be seen that most NNs associated with downstream signaling (i.e., G proteins, voltage-gated calcium channels, and protein kinases) participate in multiple systems.

Metabotropic receptor NNs are mostly located in the network core and pertain to a single system. NNs related to neurotransmitter depletion, cellular excitability, receptor-associated proteins, ionotropic receptors, neurotransmitter synthesis, and vesicle dynamics are predominantly found on the network periphery. Examples of such nodes are voltage-gated potassium channels from subfamily Q (KCNQ; especially important in nerve cells), as well as SHANK and HOMER postsynaptic density (PSD) scaffolding proteins. Although most of these nodes take part in only one neurotransmitter system, genes that encoded monoaminergic transporters (*SLC18A1* and *SLC18A2*) and oxidases (*MAOA* and *MAOB*) are present in both serotonergic and dopaminergic systems.

### NN Neuroexclusivity

**Fig. 2.**
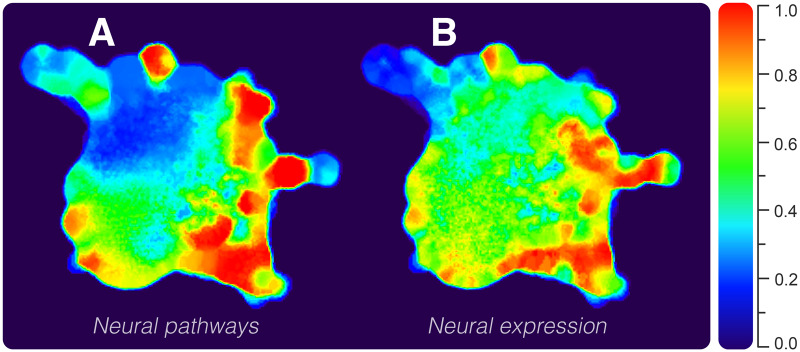
Network heatmap depicting the degree of neuroexclusivity in PPIs. The hotter the area, the more nervous system specific is the network region. Values were obtained according to genes’ (*A*) pathways and (*B*) expression in tissues.

Some NNs act exclusively in synaptic processes, whereas others are shared among basal signaling pathways in several nonnervous cell types. We quantified neuroexclusivity (i.e., how exclusive a node is to the nervous system) using annotated pathways and expression in tissues. The obtained values were superimposed on the network layout in a heatmap fashion, such that hotter regions depict highly nervous system-specific nodes (fig. 2). We computed the proportion of neural pathways for every NN (fig. 2*A*). Similarly, we also computed their proportion of neural expression (fig. 2*B*). It can be seen that both assessments agreed upon the overall distribution of neuroexclusivity, presenting higher values on the network periphery. Colder parts correspond to downstream signaling-related NNs (e.g., G protein and signaling categories), whereas hot regions correspond to ionotropic receptors, cellular excitability, receptor-associated, and vesicle-related functional categories. As observed in figure 2, neuroexclusive NNs are prone to be grouped in clusters connected to the network core by nonneuroexclusive NNs. Many of the neuroexclusive nodes connect to the whole system through down signaling cascade proteins, which are essentially nonneuroexclusive. An example is the neuroexclusive nicotinic cholinergic receptors family (*CHRN*), which connects to the network by their interaction with the tyrosine-protein kinase *JAK2*, a nonneuroexclusive protein (Bencherif and Lippiello 2010).

### Human Neurotransmission Gene Network Evolution

After evaluating which network areas are exclusively related to neural processes, we tracked the evolutionary origin of NNs. Using Clusters of Orthologous Groups (OGs) annotation present in the STRING database, we identified 88 OGs comprising the 321 NNs and determined the evolutionary root of each OG. We sought to identify the most ancient genetic archetype of each NN that has been vertically inherited during evolution (Castro et al. 2008).

**Fig. 3.**
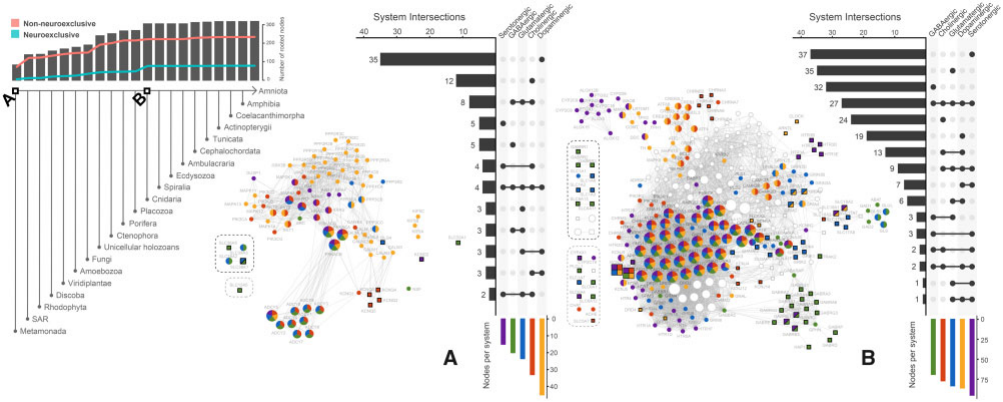
The human neurotransmission gene network filtered for nodes cumulatively rooted from the origin of eukaryotes until the Human–Cnidaria LCA. Square nodes correspond to human neuroexclusive genes. Genes are considered neuroexclusive when at least 90% of their pathways are neural pathways. Nodes enclosed by dashed lines either did not connect to the largest connected component (black dashed lines) or did not connect at all (gray dashed lines). At the top left, bars indicate the number of NNs rooted at different points of the evolutionary tree, whereas red and blue lines indicate the number of nonneuroexclusive and neuroexclusive nodes, respectively. (*A*) The network filtered for nodes rooted at the origin of eukaryotes. (*B*) The network filtered for nodes cumulatively rooted from the Eukaryota LCA to the Human–Cnidaria LCA. Nodes from *A* are colored white for comparison. Clade naming is detailed in Supplementary Material online.

According to our results, the dopaminergic system is the most widely represented neurotransmitter system in the early roots of the eukaryotic evolutionary tree. We found that half of the NNs rooted at the origin of eukaryotes (∼2 Ga [Melnikov et al. 2018]) are associated with dopaminergic synapses (fig. 3*A*). The serotonergic system is the least represented in the origin of eukaryotes, and the majority of serotonergic NNs rooted at this evolutionary point are also associated with other neurotransmitter systems in humans. Although several NNs could be traced back to the origin of eukaryotes (fig. 3*A*), the earliest metabotropic receptors were rooted at the Human–Amoebozoa LCA, belonging to the GABAB gene family. No other neurotransmitter receptor could be found until the GRM metabotropic glutamate receptor family, rooted at the Holozoa LCA (supplementary fig. 8, Supplementary Material online).

Most NNs are rooted between the origin of eukaryotes and the Human–Cnidaria LCA (fig. 3, bar chart). In fact, about 96% of NNs are rooted at the Human–Cnidaria LCA or prior to it. It is important to note that our analysis does not include prokaryotes, so the earliest point of evolutionary rooting is at the origin of eukaryotes. There is an increase of NNs rooted between the Holozoa and Human–Cnidaria LCAs (gray bars above species tree, fig. 3). Nonneuroexclusive NNs rooted at the Holozoa LCA account for most of this increase. After that, neuroexclusive NNs account for most network growth. Rooting of nonneuroexclusive NNs peaks at the Human–Porifera LCA, whereas neuroexclusive NNs rooting peaks at the Human–Cnidaria LCA. Most nodes rooted at the LCAs of early branching eukaryotes are nonneuroexclusive in humans, but some significant neuroexclusive NNs are also rooted early in evolution. For instance, *DLG4* scaffolding gene and K-Cl cotransporter *SLC12A5*, GABA and glycine transporter *SLC32A1*, glutamine transporter *SLC38A1*, amino acid transporter *SLC38A5*, potassium voltage-gated channel subfamily *KCND*, and *KCNQ* gene families, involved in neurotransmitter transport and cell excitability, are rooted at the origin of eukaryotes. However, it is not likely that *DLG4* ancient orthologs had performed the same function of the human ortholog, since the human is a multidomain protein, whereas the *Giardia intestinalis* ortholog, for instance, has only the guanylate kinase domain.

**Fig. 4.**
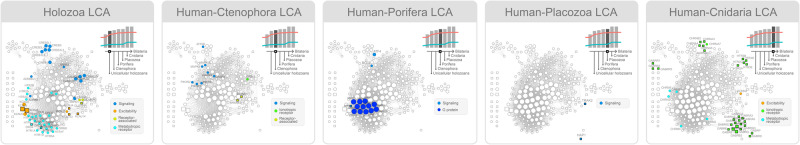
A closer look at the functional genetic archetype of the human neurotransmission network in roots prior to the establishment of anatomical synapses (assumed to have happened both at the Human–Ctenophora and Human–Cnidaria LCAs). The networks are reconstructed by cumulatively including NNs rooted from the origin of eukaryotes until each subsequent root of interest (Holozoa, Ctenophora, Porifera, Placozoa, and Cnidaria LCAs). Nodes exactly rooted at each LCA are colored according to their synaptic function, whereas nodes cumulatively rooted at previous LCAs are colored white. Square nodes correspond to human neuroexclusive genes. Genes are considered neuroexclusive when at least 90% of their pathways are neural pathways. At the top right of each panel, bars indicate the number of nodes rooted at different points of the evolutionary tree, whereas red and blue lines indicate the number of nonneuroexclusive and neuroexclusive nodes, respectively.

The majority of metabotropic receptor NNs are rooted at the Holozoa LCA. Indeed, metabotropic receptors account for half of NNs rooted in this LCA (fig. 4). It is also possible to observe the emergence of nodes related to signaling, excitability, and receptor-associated proteins at the Holozoa LCA. Our analysis is focused on proteins directly involved in neurotransmitter-based signaling. There are other molecules crucial to neuronal function such as adhesion molecules. Several of them, such as cadherins (*CDH1* and *CDH2*) and neurexins (*NRXN1*, *NRXN2*, and *NRXN3*) are also rooted at the Holozoa LCA, whereas neuroligins (*NLGN1*, *NLGN2*, *NLGN3*, and *NLGN4X*) are rooted before, at the Human–SAR LCA (supplementary table 17, Supplementary Material online). The most ancient dopamine, serotonin, and metabotropic glutamate receptors orthologs are rooted at the Holozoa LCA, although transporters for these neurotransmitters are rooted previously (e.g., the dopamine transporter *SLC6A3*, at the Human–Fungi LCA; supplementary fig. 8, Supplementary Material online). The set of metabotropic receptors is complete at the Human–Cnidaria LCA with the emergence of the first acetylcholine receptor nodes (fig. 4).

Despite the first ionotropic receptor NNs being rooted much previously at the Human–Discoba LCA (AMPA/kainate and NMDA glutamate receptor families; supplementary fig. 8, Supplementary Material online), the vast majority of ionotropic receptors are rooted at the Human–Cnidaria LCA (>600 Ma, fig. 4). Except for metabotropic acetylcholine receptors, all the NNs rooted at this evolutionary point represent ion channels and therefore exert some influence in cellular excitability. It is in agreement with previous studies in which acetylcholine receptors could only be found in cnidarians and bilaterians (Faltine-Gonzalez and Layden 2019). Besides *KCNN2*, which is activated by intracellular calcium, all NNs rooted at the Human–Cnidaria LCA represent neurotransmitter receptors from the GABAergic, cholinergic, and serotonergic systems (*GABR*, *CHRN*, *CHRM*, and *HTR3* gene families) and are found in highly neuroexclusive areas (see fig. 2). We looked for the expression of ionotropic receptor orthologs in *Nematostella vectensis* based on work developed by Sebé-Pedrós et al. (2018). Accordingly, all ionotropic receptors identified at the Human–Cnidaria LCA have orthologs expressed in *Nematostella vectensis* nervous cells (supplementary table 18, Supplementary Material online).

### Average Protein Abundance in OGs

**Fig. 5.**
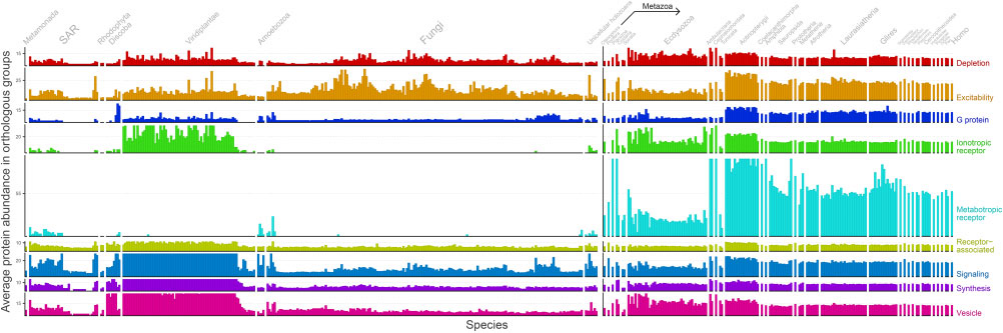
Average protein abundance in OGs across species. The horizontal axis represents organisms ordered by evolutionary relationships (supplementary fig. 1, Supplementary Material online). The rightmost organism is *Homo sapiens*, whereas the leftmost organism is *Giardia intestinalis*, its most distant relative. The vertical axis represents the average protein abundance in OGs. This axis is split and color coded according to the function of OGs, but panels still share the same scale. To aid visualization, bars were capped based on the average values of metazoan abundances (uncapped plot can be seen in supplementary fig. 10, Supplementary Material online).

Figure 5 shows the protein abundance per OG according to functional categories. It is possible to note that metabotropic and ionotropic receptors abundances are higher in metazoans when compared with unicellular holozoans. Although most metabotropic receptor NNs are rooted at the Holozoa LCA, their average abundance remains very low until *Mnemiopsis leydi*, a ctenophore. The same goes for ionotropic receptor NNs, some of which were rooted at the distant Human–Discoba LCA, despite having high abundance in iGluRs orthologs in viridiplantae. Indeed, previous researches identified iGluRs in organisms predating the divergence of animals and plants (Chiu et al. 1999). Plants are known to carry glutamate receptor-like genes (GLRs), which are homologous to the mammalian genes for iGluRs. However, mammalian iGluRs exhibit higher ligand specificity, whereas GLR receptors evolved broader nonspecific amino acid sensors, responding to as many as seven amino acids and associated with defense function (Forde and Roberts 2014).

This abundance increase in neurotransmitter receptors from metazoans could help explain the establishment of synapses observed in cnidarians and ctenophores. Additionally, there is an increased protein abundance in excitability, G protein, and signaling categories in vertebrates (from the Human–Actinopterygii LCA onwards).

## Discussion

The evolution and origin of the nervous system have always drawn widespread attention. Although several studies have been conducted looking for specific genes comparing different genomes (Dorus et al. 2004; Wu et al. 2006; de Mendoza et al. 2014; Burkhardt 2015; Krishnan and Schioth 2015), there is a gap in the understanding about the evolution of the gene interaction network of human neuronal communication. It is crucial to understand the molecular hallmark of synapse establishment since the product of many genes composing the neurotransmission network also acts in other molecular systems and evolved under different scenarios (Ryan and Grant 2009). According to our results, the nodes in the human neurotransmission network core are shared by other molecular processes, whereas those on the network periphery are mainly associated with nervous processes. Not surprisingly, core nodes involved in downstream signaling, like adenylate cyclase, and protein kinase C family, were rooted before synapse establishment, which is well described in the literature (Iseki et al. 2002; Linder and Schultz 2003). For example, adenylate cyclase enzymes are known to participate in basal environmental responses such as photoavoidance and amoebal aggregation in unicellular organisms like *Euglena gracilis* and *Dictyostelium discoideum*, respectively (Iseki et al. 2002; Linder and Schultz 2003). Protein kinases C phosphorylate several protein targets in many intracellular signaling pathways such as cell adhesion and cell cycle. It indicates that such protein families are shared between most signal transduction pathways and are not exclusively related to the five synaptic pathways evaluated here.

According to our results, half of the nodes rooted at the origin of eukaryotes are associated with the dopaminergic system. For instance, some dopaminergic gene families (e.g., calmodulin *CALM*, glycogen synthase kinase *GSK*, kinesin *KIF5*, mitogen-activated protein kinase *MAPK*, and the phosphatase proteins *PPP1* and *PPP2*) participate in cellular processes such as cell division, intracellular transport, and spatiotemporal organization of molecules and organelles (Li et al. 2011; Reddy and Day 2011; Xu et al. 2017; Saidi et al. 2012). It is clear that these dopaminergic nodes worked in other biochemical systems in the eukaryotic ancestor since synapses themselves evolved later in the evolution. Therefore, dopaminergic neurotransmission probably evolved by coopting nodes with no previous synaptic function. To some extent, this probably happened to all the five neurotransmitter systems. Previous studies demonstrated that metazoans destitute of nervous systems (e.g., sponges and placozoans) may have some synaptic components as part of cell–cell signal transmission mechanisms (Leys 2015). In fact, many genes involved in postsynaptic signaling in animals have orthologs acting in environmental responses in unicellular organisms (El-Sayed et al. 2005; Achim and Arendt 2014; Ryan 2014; Krishnan and Schioth 2015). The neurotransmitter serotonin, for instance, is broadly found in organisms lacking nervous systems (Azmitia 2007). Previous studies demonstrated that choanoflagellates endow essential factors for a primordial neurosecretory apparatus, like the Sec1/Munc18-like family, although their functions could be different in metazoans (Burkhardt et al. 2011; Burkhardt 2015). It has been hypothesized that protein machinery, such as the endocytic pathway, would allow choanoflagellates and other organisms without neurotransmission to interact with the environment (Ryan and Grant 2009). Emes and Grant (2011) reconstructed the evolutionary origin of postsynaptic proteomes of metazoan organisms by looking for domains within homologous regions conserved among Eubacteria, Archaea, and Eukaryota. The authors found that orthologs associated with basic cellular functions and responses to the external environment conserved among the three superkingdoms. On the other hand, PSD molecules critical for synapsis were absent in prokaryotic genomes (Emes and Grant 2011).

The presence of synapses in ctenophores should call our attention. Recent studies have consistently supported the placement of comb jellies as a sister group to all other animals (Moroz et al. 2014). These organisms also feature diffuse nerve nets like cnidarians, and genomic analyses indicate that their neurotransmission critically relies on ionotropic receptors (Moroz et al. 2014). Ctenophores only have iGluRs, and we found only one ionotropic receptor node rooted at the Human–Ctenophora LCA (*GRIN1*, the other glutamatergic ionotropic receptors genes were rooted at the Human–Discoba LCA). Previous studies have characterized Ctenophora iGluR orthologs as evolutionary precursors to NMDA receptors since they identified a interdomain salt bridge in the ligand-binding domain that contributes to glycine binding with higher affinity than glutamate (Alberstein et al. 2015; Yu et al. 2016).

When evaluating the whole scenario, NNs of all functions were rooted before the Human–Ctenophora LCA: neurotransmitter depletion, cellular excitability, intracellular signaling, vesicle related, metabotropic receptors, and ionotropic receptors. In other words, our results indicate that the majority of synaptic genetic elements were present before the synapse itself. The signaling improvement represented by a new ionotropic receptor could have been particularly important to the emergence of synapses in Ctenophora. Ionotropic receptors are multidomain proteins with an extracellular neurotransmitter binding site and an ion channel, directly allowing ion flow into cells upon ligand binding. Due to the direct association among neurotransmitter bind and the channel opening, ionotropic receptors are associated with a fast propagation of electrical signals (Leys 2015). Therefore, those receptors might have represented an advance in signaling speed compared with metabotropic receptors, which elicits their response through more than one metabolic step mediated by protein G cascades (Rho and Storey 2001). Except for metabotropic acetylcholine receptors rooted at the Human–Cnidaria LCA, all other metabotropic receptors were rooted before the Human–Ctenophora LCA.

The set of receptors is complete at the Human–Cnidaria LCA with the emergence of metabotropic acetylcholine receptors (CHRM family) plus the massive ionotropic receptors emergence (*GABR*, *CHRN*, and *HTR3* gene families). Therefore, this key neurotransmission mechanism has been conserved for at least 600 My. The emergence of additional receptor families, mostly ionotropic receptors, is the main network increment observed at the Human–Cnidaria LCA. Additionally, most of the nodes rooted at the Human–Cnidaria LCA are neuroexclusive in humans. Moroz and Kohn suggested that a critical number of ion channels might be necessary to support neural communication. It has been shown that species carrying neurons have a higher number of ion channels compared with nerveless species, whereas such comparisons do not hold for other functional categories (Moroz and Kohn 2015). However, ctenophores sustain synapses with only one class of ionotropic receptors (i.e., iGluRs), even that some of those iGluRs might respond to glycine (Alberstein et al. 2015; Yu et al. 2016). Additionally, placozoans do not have neurons despite having ionotropic receptor orthologs equivalent to Ctenophora. The presence of the same set of receptors in both Ctenophora and Placozoa could suggest that synapses emerged in Ctenophora and were subsequently lost in Placozoa.

Few NNs are rooted after the Human–Cnidaria LCA. The *PLA2G4* phospholipase family (seven members) is rooted at the origin of chordates and two NNs at the Human–Tunicata LCA (phosphatidylinositol 3-kinases *PIK3R5* and *PIK3R6*). Following, three nodes rooted at the origin of vertebrates deserve additional attention: *CALY*, *HOMER3*, and *PPP1R1B*. Our results converge with a previous study proposing the evolution of *CALY* gene family in the LCA of teleosts (Muthusamy et al. 2009). *CALY* encodes the endocytic transmembrane calcyon protein, playing trafficking functions primarily involved with neural development and synaptic plasticity (Davidson et al. 2009; Chander et al. 2019). *PPP1R1B* has already been associated with enhanced cognitive performance regarding frontostriatal function, neostriatal volume, and the functional connectivity of the prefrontal cortex with striatum (Meyer-Lindenberg et al. 2007). HOMER3 is part of a family of scaffolding proteins of the PSD colocalizing and modulating the activity of the metabotropic glutamate receptors (mGluR1 and mGluR5). In humans, this modulation and expression are almost exclusively present in the cerebellum, with significant implications for motor movement regulation (Höftberger et al. 2013; Ruegsegger et al. 2016). A brain anterior to the notochord with the bauplan defined by brain subdivisions is a marked innovation in vertebrates. Additionally, the evolution of jaws was crucial for active predation, guaranteeing significant advantage over filtering (Murakami et al. 2005; Roth and Dicke 2012; Šestak and Domazet-Lošo 2015; Albuixech-Crespo et al. 2017; Satizabal et al. 2019). The emergence of genes associated with motor movement regulation and coordination suggests that such increments could have played a role in the evolution of new complex feeding behavior and brain division with more specialized function during the Devonian period.

Function-specific abundances could offer insights into the evolution of nervous complexity. Vertebrates have more proteins per OGs associated with excitability, metabotropic receptor, and G protein, matching the higher complexity observed in vertebrates when compared with other organisms. In other words, the increase in abundance could have added complexity to the vertebrate network despite very few nodes being rooted after the Human–Cnidaria LCA. This suggests that some regulatory response emerged in the LCA of vertebrates by increasing synaptic paralogs. The nervous complexity observed in more recent vertebrates, like mammals, might be a consequence of the evolution of brain anatomy, such as neural architecture, the volume of cortical and subcortical areas, and the number of neurons, rather than molecular synaptic evolution. Regarding humans and other primates, additional factors must be considered for cognitive and brain evolution, such as sociality and long lifespans, that created a fruitful scenario for culture emergence (Street et al. 2017).

In summary, the genetic archetype of synaptic networks was present at the Human–Ctenophora LCA, and the network increment granted by ionotropic receptors might have been crucial to the evolution of synapses. It is challenging to precisely define the point that an ancestral ortholog starts to perform a function observed in modern organisms. The strategy presented here evaluated the whole system to identify the most probable evolutionary scenario where orthologs started to work collectively as a system. Although a greater number of synaptic orthologs in vertebrates contributed to the network complexity, it is reasonable to think that the neural architecture, rather than molecule innovation, contributed to complex neuronal phenotypes.

## Materials and Methods

### Synaptic Neurotransmission Genes Selection

Human neurotransmission genes used throughout this work were retrieved from KEGG Pathway Database API (Kanehisa and Goto 2000; Kanehisa et al. 2017) (https://www.genome.jp/kegg/, last accessed April 17, 2020) as being present in one of the following available nervous systems pathways: cholinergic synapse (hsa04725), dopaminergic synapse (hsa04728), GABAergic synapse (hsa04727), glutamatergic synapse (hsa04724), and serotonergic synapse (hsa04726), yielding a list of 325 unique Entrez Gene identifiers. Genes were manually classified according to their function in neurotransmission pathways: neurotransmitter depletion (clearance from synaptic cleft and degradation), cellular excitability, ionotropic receptors, metabotropic receptors, receptor associated, intracellular signaling, G proteins, neurotransmitter synthesis (including precursor transport), and vesicle related (supplementary table S1, Supplementary Material online). For details, please see supplementary Gene Selection and Annotation, Supplementary Material online.

### Synaptic Neurotransmission Gene Network

Entrez Gene identifiers were mapped to ENSEMBL protein and HGNC symbol identifiers and queried against STRING 11, a database that gathers direct and indirect PPIs from multiple sources (Szklarczyk et al. 2017). Three hundred and twenty-one of the initial 325 genes were found in STRING 11 and therefore remained in subsequent analysis (supplementary table S1, Supplementary Material online). PPIs were filtered for high confidence scores (≥0.7) computed from experimental and database evidence channels (von Mering et al. 2005), and then used to produce a force-directed layout (for details, please see supplementary Network, Supplementary Material online).

### Evolutionary Rooting Analysis

To address the history of genes and their corresponding neurotransmission pathways, evolutionary rooting analysis was carried out using geneplast, a Bioconductor R package developed for evolutionary analysis based on OGs distribution (Dalmolin 2015; Oliveira et al. 2019; Trefflich et al. 2020). All genes contained annotation for at least one cluster of orthologous groups (COG) such that we assume a one-to-many relationship between COG IDs (88) and genes. Geneplast determines the most probable evolutionary root of an OG by analyzing the distribution of its genes in a given species tree. In this work, we used a species tree spanning all 476 STRING eukaryotes. The tree was obtained from the TimeTree database (Kumar et al. 2017) and missing species were filled in according to NCBI Taxonomy (for details, please see supplementary Eukaryota Species Tree, Supplementary Material online).

### Neuroexclusivity

#### Neural Pathways

We manually classified KEGG pathways into two groups: neural and nonneural pathways (supplementary table S2, Supplementary Material online). To quantify neuro-exclusivity, we computed the proportion of neural pathways of each gene (supplementary table S1, Supplementary Material online). Broad base metabolism pathways “Pathways in cancer” (path: hsa05200), “Transcriptional misregulation in cancer” (path: hsa05202), and “Metabolic pathways” (path: hsa01100) were excluded from the ratio. The visualization was built using the software ViaComplex (Castro et al. 2009; Dalmolin et al. 2011) (for details, please see supplementary Pathway Neuroexclusivity, Supplementary Material online).

#### Neural Expression

All tissue-based human expression data available at EBI’s Expression Atlas (Petryszak et al. 2015) platform were collected, consisting of eight RNA-Seq projects comprising 118 tissues (E-MTAB-513, E-MTAB-2836, E-MTAB-3358, E-MTAB-3708, E-MTAB-3716, E-MTAB-4344, E-MTAB-4840, and E-MTAB-5214). Again, we manually classified tissues into two groups: nervous and nonnervous tissues. Transcripts per kilobase million gene expression across projects was aggregated into an average by tissue and then filtered for values ≥0.5. To quantify neuro-exclusivity, we computed the proportion of expression in nervous tissues of each gene. The visualization was built using the software ViaComplex (Castro et al. 2009; Dalmolin et al. 2011) (for details, please see supplementary Pathway Neuroexclusivity, Supplementary Material online).

### Average Protein Abundance in OGs

We first identified the OGs associated with each synaptic functional category (depletion, excitability, G protein, ionotropic receptor, metabotropic receptor, receptor associated, signaling, synthesis, and vesicle related). We then determined the number of proteins per OG in each functional category across the 476 species in our data set (for details, please see supplementary Abundance, Supplementary Material online).

## Supplementary Material

Supplementary data are available at *Molecular Biology and Evolution* online.

## Supplementary Material

msaa252_Supplementary_Data

## References

[msaa252-B1] Achim K , Arendt D. 2014. Structural evolution of cell types by step-wise assembly of cellular modules. Curr Opin Genet Dev. 27:102–108. 24998387 10.1016/j.gde.2014.05.001

[msaa252-B2] Aguilar JI , Dunn M, Mingote S, Karam CS, Farino ZJ, Sonders MS, Choi SJ, Grygoruk A, Zhang Y, Cela C, et al 2017. Neuronal depolarization drives increased dopamine synaptic vesicle loading via VGLUT. Neuron 95(5):1074–1088.e7. 28823729 10.1016/j.neuron.2017.07.038 PMC5760215

[msaa252-B3] Alberstein R , Grey R, Zimmet A, Simmons DK, Mayer ML. 2015. Glycine activated ion channel subunits encoded by ctenophore glutamate receptor genes. Proc Natl Acad Sci U S A. 112(44):E6048–E6057. 26460032 10.1073/pnas.1513771112 PMC4640758

[msaa252-B4] Albuixech-Crespo B , López-Blanch L, Burguera D, Maeso I, Sánchez-Arrones L, Moreno-Bravo JA, Somorjai I, Pascual-Anaya J, Puelles E, Bovolenta P, et al 2017. Molecular regionalization of the developing amphioxus neural tube challenges major partitions of the vertebrate brain. PLoS Biol. 15(4):e2001573. 28422959 10.1371/journal.pbio.2001573 PMC5396861

[msaa252-B5] Azmitia E. 2007. Cajal and brain plasticity: insights relevant to emerging concepts of mind. Brain Res Rev. 55(2):395–405. 18030688 10.1016/j.brainresrev.2007.01.010

[msaa252-B6] Belousov AB , O’Hara BF, Denisova JV. 2001. Acetylcholine becomes the major excitatory neurotransmitter in the hypothalamus in vitro in the absence of glutamate excitation. J Neurosci. 21(6):2015–2027. 11245685 10.1523/JNEUROSCI.21-06-02015.2001 PMC6762624

[msaa252-B7] Bencherif M , Lippiello PM. 2010. Alpha7 neuronal nicotinic receptors: the missing link to understanding Alzheimer’s etiopathology? Med Hypotheses 74(2):281–285. 19800174 10.1016/j.mehy.2009.09.011

[msaa252-B8] Bertozzi C , Zimmermann I, Engeler S, Hilf RJC, Dutzler R. 2016. Signal transduction at the domain interface of prokaryotic pentameric ligand-gated ion channels. PLoS Biol. 14(3):e1002393. 26943937 10.1371/journal.pbio.1002393 PMC4778918

[msaa252-B9] Bocquet N , Prado de Carvalho L, Cartaud J, Neyton J, Le Poupon C, Taly A, Grutter T, Changeux J-P, Corringer P-J. 2007. A prokaryotic proton-gated ion channel from the nicotinic acetylcholine receptor family. Nature 445(7123):116–119. 17167423 10.1038/nature05371

[msaa252-B10] Borland LM , Michael AC. 2004. Voltammetric study of the control of striatal dopamine release by glutamate. J Neurochem. 91(1):220–229. 15379902 10.1111/j.1471-4159.2004.02708.x

[msaa252-B11] Burkhardt P. 2015. The origin and evolution of synaptic proteins: choanoflagellates lead the way. J Exp Biol. 218(4):506–514. 25696814 10.1242/jeb.110247

[msaa252-B12] Burkhardt P , Stegmann CM, Cooper B, Kloepper TH, Imig C, Varoqueaux F, Wahl MC, Fasshauer D. 2011. Primordial neurosecretory apparatus identified in the choanoflagellate *Monosiga brevicollis*. Proc Natl Acad Sci U S A. 108(37):15264–15269. 21876177 10.1073/pnas.1106189108 PMC3174607

[msaa252-B13] Castro M , Rybarczyk-Filho J, Dalmolin R, Sinigaglia M, Moreira JC, Mombach JC, de Almeida R. 2009. ViaComplex: software for landscape analysis of gene expression networks in genomic context. Bioinformatics 25(11):1468–1469. 19369498 10.1093/bioinformatics/btp246

[msaa252-B14] Castro MAA , Dalmolin RJS, Moreira JCF, Mombach JCM, de Almeida RMC. 2008. Evolutionary origins of human apoptosis and genome-stability gene networks. Nucleic Acids Res. 36(19):6269–6283. 18832373 10.1093/nar/gkn636 PMC2577361

[msaa252-B15] Chander P Kennedy MJ Winckler B Weick JP. 2019. Neuron-Specific gene 2 (*NSG2*) encodes an AMPA receptor interacting protein that modulates excitatory neurotransmission. *eNeuro*. 6(1):ENEURO.0292-18.2018 10.1523/ENEURO.0292-18.2018 PMC6345199 30680309

[msaa252-B16] Chiu J , DeSalle R, Lam H, Meisel L, Coruzzi G. 1999. Molecular evolution of glutamate receptors: a primitive signaling mechanism that existed before plants and animals diverged. Mol Biol Evol. 16(6):826–838. 10368960 10.1093/oxfordjournals.molbev.a026167

[msaa252-B17] Dalmolin RJ. 2015. Geneplast: evolutionary rooting and plasticity inference. R Packag. version 1.0.0. Bioconductor. DOI: 10.18129/B9.bioc.geneplast

[msaa252-B18] Dalmolin RJ , Castro MAA, Rybarczyk Filho JL, Souza LH, de Almeida RM, Moreira JC. 2011. Evolutionary plasticity determination by orthologous groups distribution. Biol Direct 6(1):22. 21586164 10.1186/1745-6150-6-22 PMC3117832

[msaa252-B19] Davidson HT , Xiao J, Dai R, Bergson C. 2009. Calcyon is necessary for activity-dependent AMPA receptor internalization and LTD in CA1 neurons of hippocampus. Eur J Neurosci. 29(1):42–54. 19120439 10.1111/j.1460-9568.2008.06563.x PMC2771427

[msaa252-B20] de Mendoza A , Sebé-Pedrós A, Ruiz-Trillo I. 2014. The evolution of the GPCR signaling system in eukaryotes: modularity, conservation, and the transition to metazoan multicellularity. Genome Biol Evol. 6(3):606–619. 24567306 10.1093/gbe/evu038 PMC3971589

[msaa252-B21] Dorus S , Vallender EJ, Evans PD, Anderson JR, Gilbert SL, Mahowald M, Wyckoff GJ, Malcom CM, Lahn BT. 2004. Accelerated evolution of nervous system genes in the origin of Homo sapiens. Cell 119(7):1027–1040. 15620360 10.1016/j.cell.2004.11.040

[msaa252-B22] El-Sayed NM , Myler PJ, Bartholomeu DC, Nilsson D, Aggarwal G, Tran A-N, Ghedin E, Worthey EA, Delcher AL, Blandin G. 2005. The genome sequence of *Trypanosoma cruzi*, etiologic agent of Chagas disease. Science 309(5733):409–415. 16020725 10.1126/science.1112631

[msaa252-B23] Emes RD , Grant SGN. 2011. The human postsynaptic density shares conserved elements with proteomes of unicellular eukaryotes and prokaryotes. Front Neurosci. 5:44. 21503141 10.3389/fnins.2011.00044 PMC3071500

[msaa252-B24] Emes RD , Grant SGN. 2012. Evolution of synapse complexity and diversity. Annu Rev Neurosci. 35(1):111–131. 22715880 10.1146/annurev-neuro-062111-150433

[msaa252-B25] Faltine-Gonzalez DZ , Layden MJ. 2019. Characterization of nAChRs in *Nematostella vectensis* supports neuronal and non-neuronal roles in the cnidarian–bilaterian common ancestor. EvoDevo 10(1):27. 31700598 10.1186/s13227-019-0136-3 PMC6825365

[msaa252-B26] Fitzgerald GJ , Liu H, Morzorati SL. 2012. Decreased sensitivity of NMDA receptors on dopaminergic neurons from the posterior ventral tegmental area following chronic nondependent alcohol consumption. Alcohol Clin Exp Res. 36(10):1710–1719. 22433065 10.1111/j.1530-0277.2012.01762.x PMC3382036

[msaa252-B27] Forde BG , Roberts MR. 2014. Glutamate receptor-like channels in plants: a role as amino acid sensors in plant defence? F1000Prime Rep. 6:37. 24991414 10.12703/P6-37 PMC4075314

[msaa252-B28] Höftberger R , Sabater L, Ortega A, Dalmau J, Graus F. 2013. Patient with homer-3 antibodies and cerebellitis. JAMA Neurol. 70(4):506–509. 23400636 10.1001/jamaneurol.2013.1955 PMC3723144

[msaa252-B29] Iseki M , Matsunaga S, Murakami A, Ohno K, Shiga K, Yoshida K, Sugai M, Takahashi T, Hori T, Watanabe M. 2002. A blue-light-activated adenylyl cyclase mediates photoavoidance in *Euglena gracilis*. Nature 415(6875):1047–1051. 11875575 10.1038/4151047a

[msaa252-B30] Kanehisa M , Furumichi M, Tanabe M, Sato Y, Morishima K. 2017. KEGG: new perspectives on genomes, pathways, diseases and drugs. Nucleic Acids Res. 45(D1):D353–D361. 27899662 10.1093/nar/gkw1092 PMC5210567

[msaa252-B31] Kanehisa M , Goto S. 2000. KEGG: kyoto encyclopedia of genes and genomes. Nucleic Acids Res. 28(1):27–30. 10592173 10.1093/nar/28.1.27 PMC102409

[msaa252-B32] Kondziella D. 2017. The top 5 neurotransmitters from a clinical neurologist’s perspective. Neurochem Res. 42(6):1767–1771. 27822666 10.1007/s11064-016-2101-z

[msaa252-B33] Kosillo P , Zhang Y-F, Threlfell S, Cragg SJ. 2016. Cortical control of striatal dopamine transmission via striatal cholinergic interneurons. Cereb Cortex 26(11):4160–4169. 27566978 10.1093/cercor/bhw252 PMC5066833

[msaa252-B34] Krishnan A , Schioth HB. 2015. The role of G protein-coupled receptors in the early evolution of neurotransmission and the nervous system. J Exp Biol. 218(4):562–571. 25696819 10.1242/jeb.110312

[msaa252-B36] Kumar S , Stecher G, Suleski M, Hedges SB. 2017. TimeTree: a resource for timelines, timetrees, and divergence times. Mol Biol Evol. 34(7):1812–1819. 28387841 10.1093/molbev/msx116

[msaa252-B37] Leys SP. 2015. Elements of a “nervous system” in sponges. J Exp Biol. 218(4):581–591. 25696821 10.1242/jeb.110817

[msaa252-B38] Li M , Liu J, Zhang C. 2011. Evolutionary history of the vertebrate mitogen activated protein kinases family. PLoS One 6(10):e26999. 22046431 10.1371/journal.pone.0026999 PMC3202601

[msaa252-B39] Linder JU , Schultz JE. 2003. The class III adenylyl cyclases: multi-purpose signalling modules. Cell Signal. 15(12):1081–1089. 14575863 10.1016/s0898-6568(03)00130-x

[msaa252-B40] Melnikov S , Manakongtreecheep K, Söll D. 2018. Revising the structural diversity of ribosomal proteins across the three domains of life. Mol Biol Evol. 35(7):1588–1598. 29529322 10.1093/molbev/msy021 PMC5995209

[msaa252-B41] Meyer-Lindenberg A , Straub RE, Lipska BK, Verchinski BA, Goldberg T, Callicott JH, Egan MF, Huffaker SS, Mattay VS, Kolachana B, et al 2007. Genetic evidence implicating DARPP-32 in human frontostriatal structure, function, and cognition. J Clin Invest. 117(3):672–682. 17290303 10.1172/JCI30413 PMC1784004

[msaa252-B42] Momiyama T , Koga E. 2001. Dopamine D(2)-like receptors selectively block N-type Ca(2+) channels to reduce GABA release onto rat striatal cholinergic interneurones. J Physiol. 533(2):479–492. 11389206 10.1111/j.1469-7793.2001.0479a.x PMC2278623

[msaa252-B43] Moroz LL. 2015. Convergent evolution of neural systems in ctenophores. J Exp Biol. 218(4):598–611. 25696823 10.1242/jeb.110692 PMC4334147

[msaa252-B44] Moroz LL , Kocot KM, Citarella MR, Dosung S, Norekian TP, Povolotskaya IS, Grigorenko AP, Dailey C, Berezikov E, Buckley KM, et al 2014. The ctenophore genome and the evolutionary origins of neural systems. Nature 510(7503):109–114. 24847885 10.1038/nature13400 PMC4337882

[msaa252-B45] Moroz LL , Kohn AB. 2015. Unbiased view of synaptic and neuronal gene complement in ctenophores: are there pan-neuronal and pan-synaptic genes across metazoa? Integr Comp Biol. 55(6):1028–1049. 26454853 10.1093/icb/icv104 PMC4836450

[msaa252-B46] Murakami Y , Uchida K, Rijli FM, Kuratani S. 2005. Evolution of the brain developmental plan: insights from agnathans. Dev Biol. 280(2):249–259. 15882571 10.1016/j.ydbio.2005.02.008

[msaa252-B47] Muthusamy N , Ahmed SA, Rana BK, Navarre S, Kozlowski DJ, Liberles DA, Bergson C. 2009. Phylogenetic analysis of the NEEP21/calcyon/P19 family of endocytic proteins: evidence for functional evolution in the vertebrate CNS. J Mol Evol. 69(4):319–332. 19760447 10.1007/s00239-009-9273-y PMC4041480

[msaa252-B48] Negrete-Díaz JV , Sihra TS, Flores G, Rodríguez-Moreno A. 2018. Non-canonical mechanisms of presynaptic kainate receptors controlling glutamate release. Front Mol Neurosci. 11:128. 29731708 10.3389/fnmol.2018.00128 PMC5920280

[msaa252-B49] Norekian TP , Moroz LL. 2020. Comparative neuroanatomy of ctenophores: neural and muscular systems in *Euplokamis dunlapae* and related species. J Comp Neurol. 528(3):481–501. 31498892 10.1002/cne.24770

[msaa252-B50] Oliveira RA de C , de Andrade AS, Imparato DO, de Lima JGS, de Almeida RVM, Lima JPMS, Pasquali MA de B, Dalmolin RJS. 2019. Analysis of *Arabidopsis thaliana* redox gene network indicates evolutionary expansion of class III peroxidase in plants. Sci Rep. 9(1):15741. 31673065 10.1038/s41598-019-52299-y PMC6823369

[msaa252-B51] Petryszak R , Keays M, Tang YA, Fonseca NA, Barrera E, Burdett T, Füllgrabe A, Fuentes AM-P, Jupp S, Koskinen S, et al 2015. Expression Atlas update—an integrated database of gene and protein expression in humans, animals and plants. Nucleic Acids Res. 44:gkv1045. 10.1093/nar/gkv1045 PMC4702781 26481351

[msaa252-B52] Pisani D , Pett W, Dohrmann M, Feuda R, Rota-Stabelli O, Philippe H, Lartillot N, Wörheide G. 2015. Genomic data do not support comb jellies as the sister group to all other animals. Proc Natl Acad Sci U S A. 112(50):15402–15407. 26621703 10.1073/pnas.1518127112 PMC4687580

[msaa252-B53] Reddy ASN , Day IS. 2011. Microtubule motor proteins in the eukaryotic green lineage: functions and regulation. In: Liu B, editor. The plant cytoskeleton. New York: Springer. p. 119–141.

[msaa252-B54] Rho JM , Storey TW. 2001. Molecular ontogeny of major neurotransmitter receptor systems in the mammalian central nervous system: norepinephrine, dopamine, serotonin, acetylcholine, and glycine. J Child Neurol. 16(4):271–280. 11332462 10.1177/088307380101600407

[msaa252-B55] Rodríguez-Moreno A , Lerma J. 1998. Kainate receptor modulation of GABA release involves a metabotropic function. Neuron 20(6):1211–1218. 9655508 10.1016/s0896-6273(00)80501-2

[msaa252-B56] Roth G , Dicke U. 2012. Evolution of the brain and intelligence in primates. Prog Brain Res. 195:413–430. 22230639 10.1016/B978-0-444-53860-4.00020-9

[msaa252-B57] Ruegsegger C , Stucki DM, Steiner S, Angliker N, Radecke J, Keller E, Zuber B, Rüegg MA, Saxena S. 2016. Impaired mTORC1-dependent expression of homer-3 influences SCA1 pathophysiology. Neuron 89(1):129–146. 26748090 10.1016/j.neuron.2015.11.033

[msaa252-B58] Ryan JF. 2014. Did the ctenophore nervous system evolve independently? Zoology 117(4):225–226. 24986234 10.1016/j.zool.2014.06.001

[msaa252-B59] Ryan TJ , Grant SGN. 2009. The origin and evolution of synapses. Nat Rev Neurosci. 10(10):701–712. 19738623 10.1038/nrn2717

[msaa252-B60] Saidi Y , Hearn TJ, Coates JC. 2012. Function and evolution of ‘green’ GSK3/shaggy-like kinases. Trends Plant Sci. 17(1):39–46. 22051150 10.1016/j.tplants.2011.10.002

[msaa252-B61] Sakarya O , Armstrong KA, Adamska M, Adamski M, Wang I-F, Tidor B, Degnan BM, Oakley TH, Kosik KS. 2007. A post-synaptic scaffold at the origin of the animal kingdom. PLoS One 2(6):e506. 17551586 10.1371/journal.pone.0000506 PMC1876816

[msaa252-B62] Satizabal CL , Adams HHH, Hibar DP, White CC, Knol MJ, Stein JL, Scholz M, Sargurupremraj M, Jahanshad N, Roshchupkin GV, et al 2019. Genetic architecture of subcortical brain structures in 38,851 individuals. Nat Genet. 51(11):1624–1636. 31636452 10.1038/s41588-019-0511-y PMC7055269

[msaa252-B63] Sebé-Pedrós A , Saudemont B, Chomsky E, Plessier F, Mailhé M-P, Renno J, Loe-Mie Y, Lifshitz A, Mukamel Z, Schmutz S, et al 2018. Cnidarian cell type diversity and regulation revealed by whole-organism single-cell RNA-seq. Cell 173(6):1520–1534.e20. 29856957 10.1016/j.cell.2018.05.019

[msaa252-B64] Šestak MS , Domazet-Lošo T. 2015. Phylostratigraphic profiles in zebrafish uncover chordate origins of the vertebrate brain. Mol Biol Evol. 32(2):299–312. 25415965 10.1093/molbev/msu319 PMC4298178

[msaa252-B65] Silbereis JC , Pochareddy S, Zhu Y, Li M, Sestan N. 2016. The cellular and molecular landscapes of the developing human central nervous system. Neuron 89(2):248–268. 26796689 10.1016/j.neuron.2015.12.008 PMC4959909

[msaa252-B66] Street SE , Navarrete AF, Reader SM, Laland KN. 2017. Coevolution of cultural intelligence, extended life history, sociality, and brain size in primates. Proc Natl Acad Sci U S A. 114(30):7908–7914. 28739950 10.1073/pnas.1620734114 PMC5544265

[msaa252-B67] Suga H , Chen Z, de Mendoza A, Sebé-Pedrós A, Brown MW, Kramer E, Carr M, Kerner P, Vervoort M, Sánchez-Pons N, et al 2013. The Capsaspora genome reveals a complex unicellular prehistory of animals. Nat Commun. 4(1):2325. 23942320 10.1038/ncomms3325 PMC3753549

[msaa252-B68] Szklarczyk D , Morris JH, Cook H, Kuhn M, Wyder S, Simonovic M, Santos A, Doncheva NT, Roth A, Bork P, et al 2017. The STRING database in 2017: quality-controlled protein–protein association networks, made broadly accessible. Nucleic Acids Res. 45(D1):D362–D368. 27924014 10.1093/nar/gkw937 PMC5210637

[msaa252-B69] Trefflich S , Dalmolin RJS, Ortega JM, Castro MAA. 2020. Which came first, the transcriptional regulator or its target genes? An evolutionary perspective into the construction of eukaryotic regulons. Biochim Biophys Acta Gene Regul Mech. 1863(6):194472. 31825805 10.1016/j.bbagrm.2019.194472

[msaa252-B70] von Mering C , Jensen LJ, Snel B, Hooper SD, Krupp M, Foglierini M, Jouffre N, Huynen MA, Bork P. 2005. STRING: known and predicted protein–protein associations, integrated and transferred across organisms. Nucleic Acids Res. 33(Database issue):D433–D437. 15608232 10.1093/nar/gki005 PMC539959

[msaa252-B71] Wideman CE , Jardine KH, Winters BD. 2018. Involvement of classical neurotransmitter systems in memory reconsolidation: focus on destabilization. Neurobiol Learn Mem. 156:68–79. 30395938 10.1016/j.nlm.2018.11.001

[msaa252-B72] Wu Q , Gu X, Wang Y, Li N, Liu X, Wu C, Yu L, Gu X. 2006. Neurotransmitter inactivation is important for the origin of nerve system in animal early evolution: a suggestion from genomic comparison. Prog Neurobiol. 78(6):390–395. 16647180 10.1016/j.pneurobio.2006.03.002

[msaa252-B73] Xu C , Liu R, Zhang Q, Chen X, Qian Y, Fang W. 2017. The diversification of evolutionarily conserved MAPK cascades correlates with the evolution of fungal species and development of lifestyles. Genome Biol Evol. 9(2):311–322. 26957028 10.1093/gbe/evw051 PMC5381651

[msaa252-B74] Yu A , Alberstein R, Thomas A, Zimmet A, Grey R, Mayer ML, Lau AY. 2016. Molecular lock regulates binding of glycine to a primitive NMDA receptor. Proc Natl Acad Sci U S A. 113(44):E6786–E6795. 27791085 10.1073/pnas.1607010113 PMC5098608

