# Supplementary material for: Ionotropic Receptors as a Driving Force behind Human Synapse Establishment
Source: Mol Biol Evol. 2020 Sep 28;38(3):735–44. doi: 10.1093/molbev/msaa252 (PMC7947827; doi:10.1093/molbev/msaa252)
Supplement: msaa252_Supplementary_Data [file msaa252_supplementary_data.zip › supplement.pdf]

Supplementary Information

Ionotropic receptors as a driving force behind human synapse establishment

Contents

1 Project structure **1**

2 Preprocessing **2**

2.1 Eukaryota species tree 2

2.1.1 NCBI Taxonomy tree 2

2.1.2 Hybrid tree 4

2.2 Gene selection and annotation 9

2.2.1 Neurotransmitter systems annotation 9

2.2.2 Base ID lookup table 10

2.2.3 Adhesion genes 11

2.3 Neuroexclusivity 12

2.3.1 Expression neuroexclusivity 12

2.3.2 Pathway neuroexclusivity 13

2.4 Orthology data 14

2.4.1 Proteins with multiple COGs 15

2.5 Network 16

2.5.1 Retrieving network data 16

2.5.2 Recomputing scores 18

3 Analysis **19**

3.1 Root inference 19

3.1.1 Geneplast 19

3.1.2 Clade names 19

3.1.3 Phyletic patterns 21

3.1.4 Adhesion genes 24

3.2 Neuroexclusivity 25

3.2.1 Expression neuroexclusivity 25

3.2.2 Pathway neuroexclusivity 25

3.3 Network 26

3.3.1 Graph data 26
3.3.2 Manuscript figure 1 29
3.3.3 Manuscript figure 2 31
3.3.4 Manuscript figure 3 31
3.3.5 Manuscript figure 4 35
3.3.6 Supplementary network figures 36
3.3.7 Manuscript set diagrams 42
3.4 Abundance 43
3.5 Cnidarian ionotropic receptors expression 50

1 Project structure

This project is organized as an R package research compendium. Research compendia aim to provide a *"standard and easily recognizable way for organizing the digital materials of a research project to enable other researchers to inspect, reproduce, and extend the research"* (Marwick, Boettiger & Mullen, 2018). Project structure overview:

```
└──neurotransmissionevolution/
    ├──analysis/
    │   ├──plots/
    │   ├──geneplast.Rmd
    │   ├──geneplast_roots.tsv
    │   └── ...
    ├──data/
    │   ├──cogs.rda
    │   ├──gene_annotation.tsv
    │   └── ...
    ├──data-raw/
    │   ├──tree_ncbi.Rmd
    │   ├──tree_ncbi.nwk
    │   └── ...
    ├──inst/
    ├──man/
    ├──manuscripts/
    │   ├──supplement.Rmd
    │   ├──supplement.pdf
    │   └── ...
    ├──R/
    │   ├──utils.R
    │   └── ...
    ├──DESCRIPTION
    ├──NAMESPACE
    ├──neurotransmissionevolution.Rproj
    └──README.md
```

The main manuscript analysis is inside `analysis/` and uses preprocessed data from `data/`. Preprocessing steps are included in `data-raw/`. Everything is documented in RMarkdown. Some miscellaneous utility functions reside in `R/`. Additionally, both preprocessed data and utility functions are documented by roxygen2 and can be examined by usual help commands. To explore and tweak this project, users can download the repository's latest release from github (dalmolingroup/neurotransmissionevolution) and run `devtools::install()`.

2 Preprocessing

This section mainly refers to data preprocessing steps done before the actual analysis with the intent of making it either simpler or faster.

2.1 Eukaryota species tree

We opted to use the TimeTree database to obtain an standardized Eukaryota species tree. Absent species were filled in based on NCBI Taxonomy data.

2.1.1 NCBI Taxonomy tree

The following topics describe how we retrieved a target species tree from NCBI Taxonomy data.
Resources

Table 1: Lists all organisms in STRING v11.

string_species					
#	Col. name	Col. type	Used?	Example	Description
1	taxid	character	yes	9606	NCBI Taxonomy identifier
2	string_type	character	no	core	if the genome of this species is core or periphery
3	string_name	character	yes	Homo sapiens	STRING species name
4	ncbi_official_name	character	no	Homo sapiens	NCBI Taxonomy species name

Location: data-raw/download/species.v11.0.txt
Source: stringdb-static.org/download/species.v11.0.txt

Table 2: Links outdated taxon IDs to corresponding new ones.

ncbi_merged_ids					
#	Col. name	Col. type	Used?	Example	Description
1	taxid	character	yes	140100	id of node that has been merged
2	new_taxid	character	yes	666	id of node that is the result of merging

Location: data-raw/download/taxdump/merged.dmp
Source: ftp.ncbi.nlm.nih.gov/pub/taxonomy/taxdump.tar.gz

Table 3: Represents taxonomy nodes.

ncbi_edgelist					
#	Col. name	Col. type	Used?	Example	Description
1	taxid	character	yes	2	node id in NCBI taxonomy database
2	parent_taxid	character	yes	131567	parent node id in NCBI taxonomy database
3	rank	character	no	superkingdom	rank of this node
4	...	...	no	...	(too many unrelated fields)

Location: data-raw/download/taxdump/nodes.dmp
Source: ftp.ncbi.nlm.nih.gov/pub/taxonomy/taxdump.tar.gz

Table 4: Links taxon IDs to actual species names.

		`ncbi_taxon_names`			
#	Col. name	Col. type	Used?	Example	Description
1	taxid	character	yes	2	the id of node associated with this name
2	name	character	yes	Monera	name itself
3	unique_name	character	no	Monera <bacteria>	the unique variant of this name if name not unique
4	name_class	character	yes	scientific name	type of name

Location: data-raw/download/taxdump/names.dmp
Source: ftp.ncbi.nlm.nih.gov/pub/taxonomy/taxdump.tar.gz

Duplicated genera

Some species from different kingdoms may have the same genus name. Duplicated genera must be noted down because we will use them to fill in missing species at a later time.

```r
# keeping genera nodes
genera_taxids <- ncbi_edgelist %>%
  filter(rank == "genus") %>%
  select(taxid = n1, rank)

duplicated_genera <- ncbi_taxon_names %>%
  # keeping scientific names
  filter(type == "scientific name") %>%
  select(taxid = name, ncbi_name) %>%
  inner_join(genera_taxids) %>%
  # extracting and saving duplicated values
  pull(ncbi_name) %>%
  extract(duplicated(.)) %>%
  write("duplicated_genera.txt")
```

Updating STRING taxon IDs

Some organisms taxon IDs are outdated in STRING. We must update them to work with the most recent NCBI Taxonomy data.

```r
string_species %<>%
  left_join(ncbi_merged_ids) %>%
  mutate(new_taxid = coalesce(new_taxid, taxid))
```

Creating tree graph

The first step is to create a directed graph representing the NCBI Taxonomy tree.

```r
# leaving only "scientific name" rows
ncbi_taxon_names %<>%
  filter(type == "scientific name") %>%
  select(name, ncbi_name)

# finding Eukaryota taxid
eukaryota_taxon_id <- subset(ncbi_taxon_names, ncbi_name == "Eukaryota", "name", drop = TRUE)

# creating graph
g <- graph_from_data_frame(ncbi_edgelist[,2:1], directed = TRUE, vertices = ncbi_taxon_names)

# easing memory
rm(ncbi_edgelist, ncbi_merged_ids)
```

Traversing the graph

The second step is to traverse the graph from the Eukaryota root node to STRING species nodes. This automatically drops all non-eukaryotes and results in a species tree representing only STRING eukaryotes (476).

```r
eukaryote_root ← V(g)[eukaryota_taxon_id]
eukaryote_leaves ← V(g)[string_species[["new_taxid"]]]

# not_found ← subset(string_species, !new_taxid %in% ncbi_taxon_names$name)

eukaryote_paths ← shortest_paths(g, from = eukaryote_root, to = eukaryote_leaves, mode = "out")$vpath

eukaryote_vertices ← eukaryote_paths %>% unlist %>% unique

eukaryote_tree ← induced_subgraph(g, eukaryote_vertices, impl = "create_from_scratch")
```

Saving

Saving `ncbi_tree` and `string_eukaryotes` for package use. These data files are documented by the package. We also create a plain text file `476_ncbi_eukaryotes.txt` containing the updated names of all 476 STRING eukaryotes. This file will be queried against the TimeTree website.

```r
ncbi_tree ← treeio::as.phylo(eukaryote_tree)

string_eukaryotes ← string_species %>%
  filter(new_taxid %in% ncbi_tree$tip.label) %>%
  inner_join(ncbi_taxon_names, by = c("new_taxid" = "name"))

write(string_eukaryotes[["ncbi_name"]],"476_ncbi_eukaryotes.txt")

write.tree(ncbi_tree, "tree_ncbi.nwk")
usethis::use_data(string_eukaryotes, overwrite = TRUE)
```

2.1.2 Hybrid tree

Once we have both the NCBI Eukaryota tree and the list of duplicated genera, we can start assembling the complete hybrid tree.

Resources

Besides downloading all TimeTree species data (`Eukaryota_species.nwk`) we also need to manually query the website for the 476 STRING eukaryotes (`476_ncbi_eukaryotes.txt`). The file is called `476_ncbi_eukaryotes.txt` because it contains updated NCBI Taxonomy names rather than STRING outdated names. This ensures better results.

```r
download_if_missing(
  paste0("http://timetree.org/ajax/direct_download",
         "?direct-download-format=newick",
         "&direct-download-id=23070",
         "&direct-download-rank=species"),
  "Eukaryota_species.nwk"
)
```

`timetree_newick` is the tree obtained by manually uploading `476_ncbi_eukaryotes.txt` to the TimeTree website. `tree_85k` is the complete Eukaryota tree we have just downloaded.

```r
# loading species names and taxon ids
load("../data/string_eukaryotes.rda")

# loading newick tree manually obtained from timetree
timetree_newick ← read.tree("download/timetree_335_eukaryotes.nwk")

# the following genera names are unreliable and should not be searched for
duplicated_genera ← scan("duplicated_genera.txt", what = "character")

# loading all TimeTree species data we have just download (85000 species)
tree_85k ← read.tree("download/Eukaryota_species.nwk")
```

Unfound species with matching genera

Some of the 476 STRING eukaryotes are not present in the TimeTree database. However, sometimes TimeTree does contain tree data for closely related species (e.g. *Monosiga brevicollis* is not present, but *Monosiga ovata* is).

Therefore, we can use these closely related species as proxies for the actual species. This is done by searching for genera names in the complete database (`Eukaryota_species.nwk`). In the given *Monosiga brevicollis* example, we search for *Monosiga* in the complete database. We see that there is information for at least one other species of the *Monosiga* genus (in this case, *Monosiga ovata*), so we add *Monosiga brevicollis* as a sister branch to the found species.

When you search for a term in TimeTree, it uses a synonym list obtained from NCBI to try to resolve it. Sometimes TimeTree will resolve a searched term to a scientific name different from the one you searched for. The problem with this is that TimeTree does not make it obvious that it is returning a different term. The first step is to find out which species resolved to different names in the `timetree_335_eukaryotes.nwk` file:

```r
# plot(timetree_newick %>% ladderize, type = "cladogram", use.edge.length = F)

# replacing timetree species underscores with spaces
timetree_newick[["tip.label"]] %<>% str_replace_all("_", " ")

# which timetree species' names exactly match with ncbi's
taxid_indexes <- timetree_newick[["tip.label"]] %>% match(string_eukaryotes[["ncbi_name"]])

# find out which timetree species names didn't exactly match ncbi's
unmatched_names <- timetree_newick[["tip.label"]] %>% magrittr::extract(taxid_indexes %>% is.na)
print(unmatched_names)
```

```
## [1] "Cercospora fijiensis"     "Arthroderma benhamiae"
## [3] "Macropus eugenii"         "Ostreococcus lucimarinus"
## [5] "Oryza nivara"
```

```r
# manually creating lookup table to be joined
ncbi_to_timetree <- tribble(
  ~timetree_name,              ~ncbi_name,
  "Cercospora fijiensis",      "Pseudocercospora fijiensis",
  "Arthroderma benhamiae",     "Trichophyton benhamiae",
  "Macropus eugenii",          "Notamacropus eugenii",
  "Ostreococcus lucimarinus",  "Ostreococcus sp. 'lucimarinus'",
  "Oryza nivara",              "Oryza sativa f. spontanea"
)

# joining info
species_dictionary <- string_eukaryotes %>% left_join(ncbi_to_timetree)

# coalescing NAs to ncbi_name
species_dictionary %<>%
  mutate(timetree_name = coalesce(timetree_name, ncbi_name)) %>%
  mutate(timetree_name = ifelse(timetree_name %in% timetree_newick[["tip.label"]], timetree_name, NA))
```

Now we can start looking for unfound species genera in the complete tree data.

```r
# annotating genera
species_dictionary %<>%
  mutate(genus_search = coalesce(timetree_name, ncbi_name) %>%
  strsplit(" ") %>%
  sapply("[", 1))

# unique genera
selected_genera <- species_dictionary[["genus_search"]] %>% unique

# these are unreliable selected_genera:
unreliable_genera <- intersect(selected_genera, duplicated_genera)

# ensuring a cleaner newick file with only necessary data
# this is actually really important
tree_85k[["node.label"]] <- NULL
tree_85k[["edge.length"]] <- NULL

# replacing timetree's underscores with spaces
tree_85k[["tip.label"]] %<>% str_replace_all("_", " ")

# storing genus
tree_85k[["tip.genus"]] <- sapply(strsplit(tree_85k[["tip.label"]]," "), "[", 1)
```

```r
tree_85k_genera <- tree_85k[["tip.genus"]] %>% unique

# subtracting unreliable genera
tree_85k_genera %<>% setdiff(unreliable_genera)

# keeping only selected genera, including unreliable ones
tree_genus <- tree_85k %$% keep.tip(., tip.label[tip.genus %in% selected_genera])
tree_genus[["tip.genus"]] <- sapply(strsplit(tree_genus[["tip.label"]]," "), "[", 1)

# unfound species which genera are present in the 85k tree
unfound_species <- species_dictionary %>%
  filter(is.na(timetree_name) & genus_search %in% tree_85k_genera)
```

Once we figured out which species have proxy genera in the complete data, we can start filling them in as sister branches.

```r
# for each unfound species which genus is present in the 85k tree,
for(i in 1:nrow(unfound_species)){
  # we search for all species of this genus ("sister species") in the 85k tree
  # this part is tricky because bind.tip rebuilds the tree from scratch
  # so we need to keep removing underscores. there are better ways to do this.
  tip_genus <- tree_genus[["tip.label"]] %>% strsplit("[_ ]") %>% sapply("[", 1)
  sister_species <- tree_genus[["tip.label"]][tip_genus == unfound_species[[i, "genus_search"]]]
  # we obtain the sister_species' most recent common ancestor (MRCA)
  # c(.[1]) is a hack because the MRCA function only works with at least 2 nodes
  where <- getMRCA(tree_genus, sister_species %>% c(.[1]))
  # and then add a leaf node linked to this MRCA
  tree_genus %<>% bind.tip(tip.label = unfound_species[[i, "ncbi_name"]], where = where)
}

# for some reason bind.tip adds underscores to species names
tree_genus[["tip.label"]] %<>% str_replace_all("_", " ")

# keeping track of found species
found_species <- species_dictionary %>% filter(!is.na(timetree_name) | genus_search %in% tree_85k_genera)
# forced_name means it either was found in timetree or we forced it by looking at genera names
found_species %<>% mutate(forced_name = coalesce(timetree_name, ncbi_name))

# so we keep only found species in this tree we are building (timetree + forced by genera)
tree_genus %<>% keep.tip(found_species[["forced_name"]])

# which found_species rows correspond to each tip.label?
match_tiplabel_name <- match(tree_genus[["tip.label"]], found_species[["forced_name"]])

tree_genus %<>% list_modify(
# converting to ncbi taxids
  tip.label = found_species[["new_taxid"]][match_tiplabel_name]
)
```

Species of unfound genera

In this part, we try to fill in the remaining missing species (those which genera were not found in TimeTree) by searching for their closest relatives (according to NCBI Taxonomy) that are present in the current tree. Once we find its two closest relatives, we can add the missing species as a branch to their LCA. This is a conservative approach.

```r
# converting ncbi phylo to igraph
graph_ncbi <- read.tree("tree_ncbi.nwk") %>% as.igraph.phylo(directed = TRUE)

# converting phylo to igraph
graph_genus <- as.igraph.phylo(tree_genus, directed = TRUE)

# for each species which genus is not in timetree
# we'll look for its two closest species (in the NCBI tree) which are present in the tree_genus we just built
unfound_genera <- species_dictionary %>% filter(is.na(timetree_name) & !genus_search %in% tree_85k_genera)

# this is the igraph equivalent of "phylo_tree$tip.label"
tip_nodes <- V(graph_ncbi)[degree(graph_ncbi, mode = "out") == 0]

# undirected distances between all species nodes
tip_distances <- graph_ncbi %>%
  distances(v = tip_nodes, to = tip_nodes, mode = "all") %>%
  as_tibble(rownames = "from") %>%
  pivot_longer(-from, names_to = "to", values_to = "distance")
```

```r
# removing self references (zero distances)
tip_distances %<>% filter(distance > 0)

# we only want to search for species of unfound genera
tip_distances %<>% inner_join(unfound_genera %>% select(from = new_taxid))

# we only want to find species already present in the genus_tree
tip_distances %<>% inner_join(found_species %>% select(to = new_taxid))

# we only want the two closest relatives
tip_distances %<>%
  group_by(from) %>%
  top_n(-2, distance) %>% # top 2 smallest distances
  top_n(2, to) # more than 2 species have the same smallest distance, so we get the first ones

# out distance matrix between all nodes in tree, needed to find MRCAs
out_distances <- graph_genus %>% distances(mode = "out")

# for each species of unfound genera,
# we find the MRCA for its two closest relatives
unfound_genera_mrca <- tip_distances %>% group_by(from) %>% summarise(mrca = {
  # which rows have no infinite distances? the last one represents the MRCA
  mrca_row_index <- max(which(rowSums(is.infinite(out_distances[, to])) == 0))
  rownames(out_distances)[mrca_row_index]
})

# adding unfound genera species nodes
graph_genus %<>% add_vertices(nrow(unfound_genera_mrca), color = "red", attr = list(name = unfound_genera_mrca[["from"]]))

# defining unfound genera species edges
# edges_to_add[1] -> edges_to_add[2], edges_to_add[2] -> edges_to_add[3]...
edges_to_add <- V(graph_genus)[unfound_genera_mrca %>% select(mrca, from) %>% t %>% as.vector]$name

# connecting species leafs to the supposed MRCA
graph_genus %<>% add_edges(V(graph_genus)[edges_to_add])

# plotting
# plot(as.undirected(graph_genus), layout = layout_as_tree(graph_genus), vertex.label = NA, vertex.size=2)

# finally converting to phylo format
phylo_graph_genus <- treeio::as.phylo(graph_genus)

# which species_dictionary rows correspond to each tip.label?
match_tiplabel_taxid <- match(phylo_graph_genus[["tip.label"]], species_dictionary[["new_taxid"]])

phylo_graph_genus %<>% list_modify(
  # adding tip.alias (this is not exported with write.tree)
  tip.alias = species_dictionary[["string_name"]][match_tiplabel_taxid],
  # converting back to string ids
  tip.label = species_dictionary[["taxid"]][match_tiplabel_taxid]
)

# ensuring a cleaner newick file with only necessary data
phylo_graph_genus[["node.label"]] <- NULL
phylo_graph_genus[["edge.length"]] <- NULL

# usethis::use_data(phylo_graph_genus, overwrite = TRUE)
# write.tree(phylo_graph_genus, "../data/hybrid_tree.nwk")
```

Ctenophora as sister to all animals

According to TimeTree, Ctenophora remains as a sister group to Cnidaria. We believe the most recent consensus in literature is to consider them a sister group to all animals. The following code block moves *Mnemiopsis leidyi*, the only ctenophore in our analysis, to the base of the metazoan lineage.

```r
# reordering tip.labels
from_to <- c(
  "400682" = "27923",  # amphimedon to mnemiopsis
  "10228"  = "400682", # trichoplax to amphimedon
  "27923"  = "10228"   # mnemiopsis to trichoplax
)

modified_phylo <- phylo_graph_genus

modified_phylo[["tip.label"]] %<>% recode(!!!from_to)

write.tree(modified_phylo, "../data/hybrid_tree_modified.nwk")
```

Supplementary Figure 1: Complete 476 eukaryotes tree. Green species have been filled in by a genus proxy in TimeTree. Red species have been filled in by looking at NCBI Taxonomy. Clade naming is described further in this document.

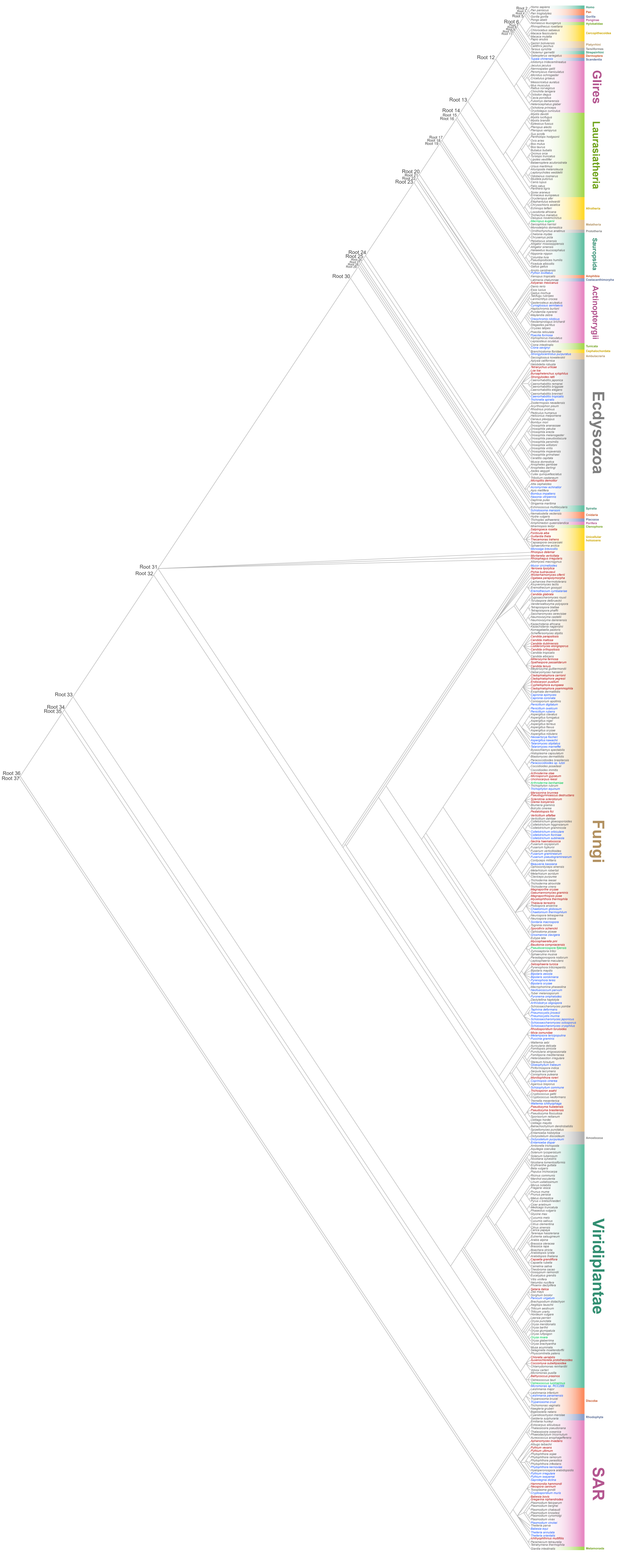

2.2 Gene selection and annotation

The anchoring point of our analysis is gene identifiers. This section describes the process of structuring an identifier dictionary as well as other useful annotation, like KEGG pathways. The goal is to have a table to which all kinds of additional data can be left joined.

2.2.1 Neurotransmitter systems annotation

We start by querying the KEGG api for the pathways of interest. Results are pivoted to a wider format.

Table 5: All links between genes and pathways in KEGG.

link_pathway_entrez					
#	Col. name	Col. type	Used?	Example	Description
1	entrez_id	character	yes	hsa:10411	NCBI Taxonomy identifier
2	pathway_id	character	yes	path:hsa04726	KEGG pathway ID

Location: data-raw/download/link_pathway_entrez.tsv
Source: http://rest.kegg.jp/link/pathway/hsa

```
pathways ← tribble(
  ~pathway_id,      ~pathway_name,
  "path:hsa04724",  "glutamatergic",
  "path:hsa04725",  "cholinergic",
  "path:hsa04726",  "serotonergic",
  "path:hsa04727",  "gabaergic",
  "path:hsa04728",  "dopaminergic"
)

# Removing hsa prefix
link_pathway_entrez[["entrez_id"]] %<>% str_split_n(":", 2)

# Filtering for pathways of interest and pivoting
gene_pathways ← inner_join(link_pathway_entrez, pathways) %>%
  mutate(n = 1) %>%
  pivot_wider(
    id_cols     = entrez_id,
    names_from  = pathway_name,
    values_from = n,
    values_fn   = list(n = length),
    values_fill = list(n = 0)
  ) %>%
  mutate(system_count = rowSums(select(., -entrez_id)))

# Exporting for package use
usethis::use_data(gene_pathways, overwrite = TRUE)
```

tail(gene_pathways)						
entrez_id	glutamatergic	cholinergic	serotonergic	gabaergic	dopaminergic	system_count
805	0	0	0	0	1	1
808	0	0	0	0	1	1
810	0	0	0	0	1	1
84152	0	0	0	0	1	1
91860	0	0	0	0	1	1
9575	0	0	0	0	1	1

2.2.2 Base ID lookup table

Now we start building a base ID lookup table containing entrez gene IDs, STRING ensembl protein IDs, ensembl gene IDs, STRING protein names and entrez gene names. Every piece of data in subsequent analyses will be progressively joined to it.

Table 6: Conversion dictionary from entrez ID to STRING's ensembl protein ID.

link_entrez_string					
#	Col. name	Col. type	Used?	Example	Description
1	taxid	numeric	no	9606	NCBI Taxonomy ID
2	entrez_id	numeric	yes	7157	entrez gene ID
3	string_id	character	yes	9606.ENSP00000269305	STRING ID

Location: data-raw/download/human.entrez_2_string.2018.tsv.gz
Source: https://string-db.org/mapping_files/entrez/human.entrez_2_string.2018.tsv.gz

Table 7: Conversion dictionary from STRING ID to protein name.

string_names					
#	Col. name	Col. type	Used?	Example	Description
1	taxid	numeric	no	9606	NCBI Taxonomy ID
2	string_name	character	yes	TP53	protein name
3	string_id	character	yes	9606.ENSP00000269305	STRING ID

Location: data-raw/download/human.name_2_string.tsv.gz
Source: https://string-db.org/mapping_files/STRING_display_names/human.name_2_string.tsv.gz

Table 8: Conversion dictionary from entrez ID to gene name.

entrez_names					
#	Col. name	Col. type	Used?	Example	Description
1	taxid	numeric	no	9606	taxon ID
2	entrez_id	character	yes	7157	entrez gene ID
3	entrez_name	character	yes	TP53	gene name
4	...	...	no	...	(too many unrelated fields)

Location: data-raw/download/Homo_sapiens.gene_info.gz
Source: https://ftp.ncbi.nlm.nih.gov/gene/DATA/GENE_INFO/Mammalia/Homo_sapiens.gene_info.gz

Table 9: Conversion dictionary from entrez ID to ensembl gene (ENSG) ID.

link_ensembl_entrez					
#	Col. name	Col. type	Used?	Example	Description
1	entrez_id	character	yes	hsa:7157	entrez gene ID
2	ensembl_id	character	yes	ensembl:ENSG00000141510	ensembl gene ID

Location: data-raw/download/link_ensembl_entrez.tsv
Source: http://rest.genome.jp/link/ensembl/hsa

```r
# Removing all kegg prefixes (e.g. "hsa:")
link_ensembl_entrez %<>% mutate_all(str_split_n, ":", 2)

# Joining all data
gene_ids <- gene_pathways %>%
  select(entrez_id) %>%
  left_join(link_ensembl_entrez) %>%
  left_join(link_entrez_string) %>%
  left_join(string_names) %>%
  left_join(entrez_names)
```

Some STRING proteins couldn't be automatically resolved, so we resolve them manually

```r
gene_ids[!complete.cases(gene_ids),]
```

entrez_id	ensembl_id	string_id	string_name	entrez_name
100137049	ENSG00000243708	NA	NA	PLA2G4B
85358	ENSG00000251322	NA	NA	SHANK3
8681	ENSG00000168970	NA	NA	JMJD7-PLA2G4B
1139	ENSG00000175344	NA	NA	CHRNA7
107987478	NA	NA	NA	LOC107987478
107987479	NA	NA	NA	LOC107987479
1564	ENSG00000205702	NA	NA	CYP2D7
801	ENSG00000198668	NA	NA	CALM1
805	ENSG00000143933	NA	NA	CALM2
808	ENSG00000160014	NA	NA	CALM3

```r
complete_info <- tribble(
##########################################################################################
 ~entrez_id,       ~ensembl_id,             ~string_id,    ~string_name,    ~entrez_name,#
"100137049", "ENSG00000243708", "9606.ENSP00000396045",       "PLA2G4B",       "PLA2G4B",#
    "85358", "ENSG00000251322",                     NA,              NA,        "SHANK3",#
     "8681", "ENSG00000168970", "9606.ENSP00000371886", "JMJD7-PLA2G4B", "JMJD7-PLA2G4B",#
     "1139", "ENSG00000175344", "9606.ENSP00000407546",        "CHRNA7",        "CHRNA7",#
"107987478",                NA,                     NA,              NA,  "LOC107987478",#
"107987479",                NA,                     NA,              NA,  "LOC107987479",#
     "1564", "ENSG00000205702",                     NA,              NA,        "CYP2D7",#
      "801", "ENSG00000198668", "9606.ENSP00000349467",         "CALM1",         "CALM1",#
      "805", "ENSG00000143933", "9606.ENSP00000272298",         "CALM2",         "CALM2",#
      "808", "ENSG00000160014", "9606.ENSP00000291295",         "CALM3",         "CALM3" #
)#########################################################################################

# Removing incomplete cases and adding updated ones
gene_ids %<>% na.omit %>% bind_rows(complete_info)

# Removing taxid prefix from STRING IDs
gene_ids[["string_id"]] %<>% str_split_n("\\.", 2)

# Exporting for package use
usethis::use_data(gene_ids, overwrite = TRUE)
```

2.2.3 Adhesion genes

Further in this document we perform an additional analysis in which the roots of neural adhesion genes are inferred. The following blocks build a separate identifier dictionary for these genes. We start by scraping the "Cell adhesion molecules" KEGG pathway page for the "Neural system"-related genes:

```r
adhesion_html <- read_html("https://www.genome.jp/kegg-bin/show_pathway?hsa04514")

# Scraping KEGG for neural adhesion genes
neural_system <- adhesion_html %>%
  html_nodes('area[shape="rect"][href^="/dbget-bin/www_bget?hsa:"]') %>%
  keep(~ {
```

```r
    x_coord <- html_attr(.x, "data-coords") %>% strsplit(",") %>% map(1) %>% as.numeric
    # The neural system-related genes are located between x coordinates 800 and 1200
    return(x_coord >= 800 & x_coord <= 1200)
  }) %>%
  {
    entrez_id <- html_attr(., "href") %>% strsplit("\\?") %>% map_chr(2) %>% strsplit("\\+") %>% unlist
    title     <- html_attr(., "title") %>% strsplit(", ") %>% unlist
    data.frame(entrez_id = entrez_id, title = title)
  } %>%
  distinct

adhesion_genes <- neural_system %>%
  transmute(
     entrez_id    = entrez_id %>% str_split(":") %>% map_chr(2)
    ,pathway_name = "adhesion"
  )
```

Building the base ID lookup table:

```r
adhesion_genes %<>% left_join(link_entrez_string) %>% left_join(entrez_names)
```

One protein was not found in STRING:

```r
adhesion_genes[!complete.cases(adhesion_genes),"entrez_name"]
```

```
## [1] "NLGN4Y"
```

Exporting data for analysis:

```r
# Removing unfound proteins
adhesion_genes %<>% na.omit

# Removing taxid prefix from STRING IDs
adhesion_genes[["string_id"]] %<>% str_split("\\.") %>% map_chr(2)

# Exporting for package use
usethis::use_data(adhesion_genes, overwrite = TRUE)
```

2.3 Neuroexclusivity

Neuroexclusivity data consists of gene expression collected from Gene Expression Atlas and the KEGG pathways themselves. This data is cleaned and subsetted prior to analysis.

2.3.1 Expression neuroexclusivity

Multiple wide .tsv files are preprocessed into a single long data frame. We also create a template file for manually classifying tissues into nervous or non-nervous categories.

Resources
We start by searching Gene Expression Atlas for experiments that have human baseline expression data at the tissue level. For each experiment, TPM expression data is downloaded to the `data-raw/download/gxa/` directory. The following 8 experiments could be found (hyperlinked):

- E-MTAB-513
- E-MTAB-2836
- E-MTAB-3358
- E-MTAB-3708
- E-MTAB-3716

- E-MTAB-4344
- E-MTAB-4840
- E-MTAB-5214

Reshaping

Loading and pivoting all data to a long format.

```r
# Loading
gene_expression <- sapply(
  list.files("download/gxa/", full.names = T),
  read_tsv,
  comment = "#",
  simplify = FALSE,
  USE.NAMES = TRUE
)

# Pivoting
gene_expression %<>%
  map_dfr(pivot_longer, cols = -(1:2), names_to = "tissue", values_to = "tpm") %>%
  na.omit %>%
  select(ensembl_id = `Gene ID`, tissue, tpm)
```

Cleaning

A lot of tissue annotation can be collapsed into single levels (e.g. “brain” and “brain fragment” can be considered the same tissue). The cleaning is performed and expression data is exported for analysis.

```r
# E-MTAB-4840 has comma separated developmental stage info (removing everything before ", ")
gene_expression %<>% mutate(tissue = str_remove(tissue, "^.+, "))

tissue_names_fix <- c(
  "brain fragment"                      = "brain",
  "forebrain fragment"                  = "forebrain",
  "forebrain and midbrain"              = "forebrain",
  "hindbrain fragment"                  = "hindbrain",
  "hindbrain without cerebellum"        = "hindbrain",
  "hippocampus proper"                  = "hippocampus",
  "hippocampal formation"               = "hippocampus",
  "diencephalon and midbrain"           = "diencephalon",
  "visceral (omentum) adipose tissue"   = "adipose tissue",
  "subcutaneous adipose tissue"         = "adipose tissue",
  "spinal cord (cervical c-1)"          = "spinal cord",
  "C1 segment of cervical spinal cord"  = "spinal cord"
)

gene_expression %<>% mutate(tissue = recode(tissue, !!!tissue_names_fix))

# Subseting for genes of interest
gene_expression %<>% filter(ensembl_id %in% gene_ids[["ensembl_id"]])

# Exporting for package use
usethis::use_data(gene_expression, overwrite = TRUE)
```

Tissue classification

For subsequent analyses, we need to distinguish if a tissue is part of the nervous system or not. This is done by hand. The first step is to write a temp file to `data-raw/temp/temp_tissue_classification.tsv` with all tissue names. This serves as a base for the completed `data/neuroexclusivity_classification_tissue` file.

```r
gene_expression %>%
  select(tissue) %>%
  unique %>%
  arrange %>%
  mutate(is_nervous = NA) %>%
  write_tsv("temp/temp_tissue_classification.tsv")
```

2.3.2 Pathway neuroexclusivity

In this section we create a template file for classifying pathways into nervous or non-nervous.

Resources For `link_pathway_entrez` see Table 5.

Table 10: KEGG pathway names.

pathway_names					
#	Col. name	Col. type	Used?	Example	Description
1	pathway_id	character	yes	path:hsa04726	KEGG pathway ID
2	pathway_name	character	yes	Serotonergic synapse - Homo sapiens (human)	pathway name

Location: data-raw/download/pathway_names.tsv
Source: http://rest.kegg.jp/list/pathway/hsa

Pathway classification
Just like tissues, we need to distinguish if a pathway is related to the nervous system or not. This is done by hand. The first step is to write a temp file to `data-raw/temp/temp_pathway_classification.tsv` with all pathway names. This serves as a base for the completed `data/neuroexclusivity_classification_pathway.tsv` file.

```
# Removing species prefix ("hsa:")
link_pathway_entrez[["entrez_id"]] %<>% str_split_n("\\:", 2)

selected_genes_pathways ← link_pathway_entrez %>% filter(entrez_id %in% gene_ids[["entrez_id"]])

unique_pathway_ids ← selected_genes_pathways %>% pull(pathway_id) %>% unique

pathway_names %<>% filter(pathway_id %in% unique_pathway_ids) %>%
  mutate(is_nervous = NA) %>%
  write_tsv("temp/temp_pathway_classification.tsv")
```

2.4 Orthology data

This section refers to orthology data exported for geneplast use. Essentially, we subset the global STRING mapping between proteins and orthologous groups into a smaller dataset containing only information about orthogroups related to eukaryotic species.

Table 11: Orthologous groups (COGs, NOGs, KOGs) and their proteins.

cogs					
#	Col. name	Col. type	Used?	Example	Description
1	taxid.string_id	character	yes	9606.ENSP00000269305	STRING protein ID
2	start_position	numeric	no	1	residue where orthogroup mapping starts
3	end_position	numeric	no	393	residue where orthogroup mapping ends
4	cog_id	character	yes	NOG08732	orthologous group ID
5	protein_annotation	character	no	Cellular tumor antigen p53; [...]	protein description

Location: data-raw/download/COG.mappings.v11.0.txt.gz
Source: https://stringdb-static.org/download/COG.mappings.v11.0.txt.gz

```
# Spliting first column into taxid and string_id
separated_ids ← cogs %$% stri_split_fixed(taxid.string_id, pattern = ".", n = 2, simplify = T)

cogs[["taxid"]]     ← separated_ids[, 1]
cogs[["string_id"]] ← separated_ids[, 2]

# Freeing up some memory
rm(separated_ids)
gc()

# keeping only eukaryotes
cogs %<>% select(-taxid.string_id) %>% filter(taxid %in% string_eukaryotes[["taxid"]])
gc()
```

```r
# Subsetting cogs of interest
gene_cogs <- cogs %>%
    filter(string_id %in% gene_ids[["string_id"]]) %>%
    select(-taxid) %>%
    group_by(string_id) %>%
    summarise(n = n(), cog_id = paste(cog_id, collapse = " / "))

# Subsetting adhesion cogs of interest
gene_cogs_extra <- cogs %>%
    filter(string_id %in% adhesion_genes[["string_id"]]) %>%
    select(-taxid) %>%
    group_by(string_id) %>%
    summarise(n = n(), cog_id = paste(cog_id, collapse = " / "))
```

2.4.1 Proteins with multiple COGs

Some proteins are occasionally assigned to more than one orthologous group (OG) by the COG algorithm. Despite infrequent, a decision needs to be made to deal with these exceptions. Since we aim to identify the most ancient archetype that has been vertically inherited to humans, it is reasonable to resolve these proteins to their most ancient OG (in the case of a human protein assigned to more than one OG). However, this strategy can sometimes be inappropriate for multidomain proteins. For instance, the SHANK proteins are assigned to two OG, of which the most ancient one is simply associated with ankyrin repeats. A similar case involves the PLA2G4 family and C2 domains, where the six members are assigned to more than one OG. We decided to resolve SHANK and PLA2G4 proteins to their most recent OGs.

```r
gene_cogs %>% filter(n > 1)
```

Table 12: Proteins assigned to more than one OG.

string_id	n	cog_id
ENSP00000290472	2	KOG1028 / KOG1325
ENSP00000293441	2	COG0666 / KOG4375
ENSP00000356436	2	COG5038 / KOG1325
ENSP00000371886	3	COG1226 / KOG1028 / KOG1325
ENSP00000380442	2	KOG1028 / KOG1325
ENSP00000382434	2	KOG1028 / KOG1325
ENSP00000396045	2	KOG1028 / KOG1325
ENSP00000469689	2	COG0666 / KOG4375

```r
gene_cogs_extra %>% filter(n > 1)
```

Table 13: One Adhesion protein has been assigned to more than one OG.

string_id	n	cog_id
ENSP00000359085	2	KOG1836 / KOG3512

Manually resolving them to their most recent OG (which was determined in a previous run).

```r
# Resolving main proteins
gene_cogs_resolved <- tribble(
       ~string_id,   ~cog_id,
"ENSP00000371886", "KOG1325",# JMJD7-PLA2G4B
"ENSP00000356436", "KOG1325",# PLA2G4A
"ENSP00000396045", "KOG1325",# PLA2G4B
```

```
"ENSP00000290472", "KOG1325",# PLA2G4D
"ENSP00000382434", "KOG1325",# PLA2G4E
"ENSP00000380442", "KOG1325",# PLA2G4F
"ENSP00000293441", "KOG4375",# SHANK1
"ENSP00000469689", "KOG4375",# SHANK2
)

# Removing unresolved cases and adding manual assignments
gene_cogs %<>%
  filter(n == 1) %>%
  select(-n) %>%
  bind_rows(gene_cogs_resolved)

# Resolving extra adhesion proteins
gene_cogs_extra_resolved <- tribble(
       ~string_id,    ~cog_id,
"ENSP00000359085", "KOG3512",# NTNG1
)

# Removing unresolved cases and adding manual assignments
gene_cogs_extra %<>%
  filter(n == 1) %>%
  select(-n) %>%
  bind_rows(gene_cogs_extra_resolved)
```

Exporting for analysis:

```
# Exporting for package use
usethis::use_data(cogs, overwrite = TRUE)
usethis::use_data(gene_cogs, overwrite = TRUE)
usethis::use_data(gene_cogs_extra, overwrite = TRUE)
```

2.5 Network

We use our gene dictionary to retrieve the corresponding human protein-protein interaction network. This is done through the STRING API. In the end, we recompute combined interaction scores.

2.5.1 Retrieving network data

Querying the API endpoint for the STRING IDs we previously resolved:

```
identifiers <- gene_ids %>% pull(string_id) %>% na.omit %>% paste0(collapse="%0d")

if (!file.exists("download/string_ids.tsv")) {
    postForm(
      "http://string-db.org/api/tsv/get_string_ids"
      ,identifiers = identifiers
      ,echo_query  = "1"
      ,species     = "9606"
    ) %>%
    write("download/string_ids.tsv")
}
```

Table 14: STRING interaction network with channel specific scores.

string_ids					
#	Col. name	Col. type	Used?	Example	Description
1	queryItem	character	yes	ENSP00000258400	queried term
2	queryIndex	numeric	yes	266	index of queried term
3	stringId	character	yes	9606.ENSP00000258400	STRING ID
4	ncbiTaxonId	numeric	yes	9606	NCBI Taxonomy ID
5	taxonName	character	yes	Homo sapiens	species name
6	preferredName	character	yes	HTR2B	common protein name
7	annotation	character	yes	5-hydroxytryptamine receptor 2B; [...]	protein annotation

Location: data-raw/download/string_ids.tsv
Source: http://string-db.org/api/tsv/get_string_ids

Making sure that the API succesfully resolves queried protein IDs.

```
api_ids ← read_tsv("download/string_ids.tsv", comment = "", quote = "")

# Removing taxid prefix
api_ids %<>% mutate(stringId = str_split_n(stringId, "\\.", 2))

# Removing inexact matches (queried id is different from resolved id)
api_ids %<>% group_by(queryItem) %>% filter(queryItem == stringId)

# Setequal must return true if ids matched exatcly
setequal(
  gene_ids %>% pull(string_id) %>% na.omit,
  api_ids  %>% pull(stringId)
) %>% stopifnot
```

Once IDs are correctly resolved, we can query the network API endpoint to obtain the protein-protein interaction edgelist.

```
# It is important to query this endpoint with the species prefix ("9606.")
identifiers ← api_ids %>% pull(stringId) %>% na.omit %>% { paste0("9606.", ., collapse="%0d") }

if (!file.exists("download/string_edgelist.tsv")) {
    postForm(
      "http://string-db.org/api/tsv/network"
      ,identifiers = identifiers
      ,species     = "9606"
    ) %>%
    write("download/string_edgelist.tsv")
}
```

Table 15: STRING interaction network with channel specific scores.

string_edgelist					
#	Col. name	Col. type	Used?	Example	Description
1	stringId_A	character	yes	ENSP00000215659	STRING ID (protein A)
2	stringId_B	character	yes	ENSP00000211287	STRING ID (protein B)
3	preferredName_A	character	yes	MAPK12	common protein name (protein A)
4	preferredName_B	character	yes	MAPK13	common protein name (protein B)
5	ncbiTaxonId	numeric	yes	9606	NCBI Taxonomy ID
6	score	numeric	yes	0.948	combined score
7	nscore	numeric	yes	0	gene neighborhood score
8	fscore	numeric	yes	0	gene fusion score
9	pscore	numeric	yes	0.014223	phylogenetic profile score
10	ascore	numeric	yes	0	coexpression score
11	escore	numeric	yes	0.485	experimental score
12	dscore	numeric	yes	0.9	database score
13	tscore	numeric	yes	0.02772	textmining score

Location: data-raw/download/string_edgelist.tsv
Source: http://string-db.org/api/tsv/network

2.5.2 Recomputing scores

From string-db.org:

> "In STRING, each protein-protein interaction is annotated with one or more 'scores'. Importantly, these scores do not indicate the strength or the specificity of the interaction. Instead, they are indicators of confidence, i.e. how likely STRING judges an interaction to be true, given the available evidence. All scores rank from 0 to 1, with 1 being the highest possible confidence."

For the sake of this project, we will only use experimental and database scores with a combined value >= 0.7, a high confidence threshold according to the STRING database. The combined score is given by the following expression, as stated in von Mering C et al, 2005:

$$S = 1 - \prod_i (1 - S_i)$$

```r
string_edgelist <- read_tsv("download/string_edgelist.tsv")

string_edgelist %<>%
  mutate(cs = combine_scores(., c("e","d"))) %>%
  filter(cs >= 0.7) %>%
  select(stringId_A, stringId_B)

# How many edgelist proteins are absent in gene_ids? (should return 0)
setdiff(
  string_edgelist %$% c(stringId_A, stringId_B),
  gene_ids %>% pull(string_id)
) %>% { stopifnot(. == 0) }

# Exporting for package use
usethis::use_data(string_edgelist, overwrite = TRUE)
```

3 Analysis

This section focuses on analyses and results reported in the main manuscript.

3.1 Root inference

To estimate the evolutionary root of a given gene (i.e. the ancestor from which its genetic archetype was vertically inherited), we use orthologous group annotation from the STRING database. The presence and absence of an orthologous group in a species tree is used to determine its most likely evolutionary root. Loading initial resources:

```
library(tidyverse)
library(magrittr)
library(geneplast)
library(ape)
library(XML)
library(rentrez)
library(neurotransmissionevolution)

data(
  cogs,
  gene_cogs,
  string_eukaryotes,
  package = "neurotransmissionevolution"
)

phyloTree <- read.tree("../data/hybrid_tree_modified.nwk") %>% rotatePhyloTree("9606")
```

We perform some minor data formatting before feeding it to geneplast

```
# Formating cogdata column names for geneplast
cogs %<>% select(protein_id = string_id, ssp_id = taxid, cog_id)

# Adding species names to taxid tree
phyloTree %<>% list_modify(
  tip.alias = string_eukaryotes %$% string_name[match(phyloTree[["tip.label"]], taxid)]
)
```

3.1.1 Geneplast

Geneplast's `groot.preprocess` function structures an `ogr` object on which `groot` will perform the rooting. We then retrieve the numeric root (`groot.get("results")`) for the `cogs_of_interest`, that is, orthologous groups pertaining to neurotransmission genes.

```
cogs_of_interest <- gene_cogs %>% pull(cog_id) %>% unique

ogr <- groot.preprocess(
  cogdata   = cogs,
  phyloTree = phyloTree,
  spid      = "9606",
  cogids    = cogs_of_interest
)

roots <- groot(ogr, nPermutations = 1) %>%
  groot.get("results") %>%
  rownames_to_column("cog_id") %>%
  select(cog_id, root = Root) %>%
  write_tsv("geneplast_roots.tsv")
```

3.1.2 Clade names

Each root branches to a clade that diverged from humans some time in the past. It is nice to have these clades taxonomically named to ease our interpretation. Unlike NCBI Taxonomy, TimeTree's internal nodes are not named.

Therefore, we query the NCBI Taxonomy API to try to find most clade names automatically. It is important to note that we are using a hybrid tree primarily built from TimeTree data. This means NCBI Taxonomy naming will not perfectly match clades in our tree. For instance, root #36 branches to a clade containing 38 species from the SAR supergroup, but also 1 species from the Haptista rank, namely *Emiliania huxleyi*. The Haptista group is a sister clade to SAR, so it might be the case that *Emiliania huxleyi* is actually correctly placed together with SAR species by TimeTree, given their evolutionary proximity. Resolving these naming conflicts is not trivial and falls out of our scope.

```r
# Querying NCBI Taxonomy with our taxids
lineages <- entrez_fetch(
  db      = "taxonomy",
  id      = string_eukaryotes[["new_taxid"]],
  rettype = "xml",
  retmode = "xml",
  parsed  = TRUE
)

# Parsing the XML result and retrieving lineage data
string_eukaryotes %<>% mutate(
  root        = ogr@tree$tip.group[taxid],
  lineage_txt = xpathSApply(lineages, "//Lineage", XML::xmlValue)
)

# Writing all lineage data to manually check for edge cases
string_eukaryotes %>%
  select(root, lineage_txt) %>%
  arrange(root, lineage_txt) %>%
  write_tsv("temp/species_lineage.txt")

# The following chain of dplyr verbs
# is responsible for figuring out the best clade names
root_names <- string_eukaryotes %>%

  # Long format lineages
  mutate(
     lineage_split = strsplit(lineage_txt, "; ")
  ) %>%
  unnest_longer(
     col         = lineage_split
    ,values_to  = "clade_name"
    ,indices_to = "clade_depth"
  ) %>%

  # Counting and dropping the last group
  group_by(root, clade_depth, clade_name) %>%
  tally(sort = TRUE) %>%

  # Collapsing lineages by clade depths
  summarise(
     diverging_rank = n_distinct(clade_name) > 1
    ,clade_name     = ifelse(diverging_rank, paste0(clade_name, " (", n,")", collapse = "; "), clade_name)
  ) %>%

  # Removing diverging ranks after the first one
  filter(cumsum(diverging_rank) <= 1) %>%

  # Removing irrelevant basal ranks (eg Eukaryota)
  group_by(clade_depth) %>%
  arrange(root) %>%
  filter(!(duplicated(clade_name) | duplicated(clade_name, fromLast = TRUE)) | diverging_rank) %>%

  # Choosing name
  group_by(root) %>%
  summarise(clade_name = first(clade_name, order_by = clade_depth)) %>%
  write_tsv("temp/temp_geneplast_clade_names.tsv")
```

Some automatically named clades have to be resolved by hand. The following table shows clade names before and after manual checking:

Table 16: Clade names before and after manual checking.

Root	Automatic name	Corrected name
1	Homo	Homo
2	Pan	Pan
3	Gorilla	Gorilla
4	Ponginae	Ponginae
5	Hylobatidae	Hylobatidae
6	Cercopithecoidea	Cercopithecoidea
7	Platyrrhini	Platyrrhini
8	Tarsiiformes	Tarsiiformes
9	Strepsirrhini	Strepsirrhini
10	Dermoptera	Dermoptera
11	Scandentia	Scandentia
12	Glires	Glires
13	Laurasiatheria	Laurasiatheria
14	Afrotheria (6); Xenarthra (1)	Afrotheria
15	Metatheria	Metatheria
16	Prototheria	Prototheria
17	Sauropsida	Sauropsida
18	Amphibia	Amphibia
19	Coelacanthimorpha	Coelacanthimorpha
20	Actinopterygii	Actinopterygii
21	Tunicata	Tunicata
22	Cephalochordata	Cephalochordata
23	Echinodermata (1); Hemichordata (1)	Ambulacraria
24	Ecdysozoa (43); Spiralia (2)	Ecdysozoa
25	Spiralia	Spiralia
26	Cnidaria	Cnidaria
27	Placozoa	Placozoa
28	Porifera	Porifera
29	Ctenophora	Ctenophora
30	Opisthokonta (5); Apusozoa (1); Cryptophyceae (1)	Unicellular holozoans
31	Fungi	Fungi
32	Amoebozoa	Amoebozoa
33	Viridiplantae	Viridiplantae
34	Discoba (6); Metamonada (1); Sar (1)	Discoba
35	Rhodophyta	Rhodophyta
36	Sar (38); Haptista (1)	SAR
37	Metamonada	Metamonada

3.1.3 Phyletic patterns

Visualizing the presence/absence of COGs across species.

```
lca_names %<>% rename("lca" = root)

lca_spp <- ogr@spbranches %>%
  rename("taxid" = ssp_id, "species" = ssp_name, "lca" = `9606`) %>%
  mutate(taxid_order = row_number())

# Saving for use in abundance computation
lca_spp %>% select(lca, taxid, taxid_order) %>% write_tsv("geneplast_clade_taxids.tsv")

cog_pam <- ogr@orthoct[,-1]

long_pam <- cog_pam %>%
  rownames_to_column("taxid") %>%
```

```r
  pivot_longer(-taxid, names_to = "cog_id") %>%
  left_join(lca_spp) %>%
  left_join(lca_names) %>%
  left_join(roots) %>%
  mutate(
    cog_id       = fct_reorder(cog_id, root),
    species      = fct_reorder(species, desc(taxid_order)),
    clade_name   = fct_reorder(clade_name, lca),
    root         = as_factor(root),
    clade_stripe = as.numeric(as_factor(lca)) %% 2 == 0
  ) %>%
  # Stripe every other species
  group_by(cog_id) %>%
  mutate(spp_stripe = as.numeric(species) %% 2 == 0) %>%
  # Removing empty tiles
  filter(value == 1) %>%
  # Stripe every other cog
  group_by(taxid) %>%
  mutate(cog_stripe = as.numeric(cog_id) %% 2 == 0)

ggplot(long_pam, aes(x = cog_id, y = species)) +
  geom_tile(aes(fill = clade_stripe + 0.3 * xor(spp_stripe, cog_stripe))) +
  scale_fill_gradient(low = "#424242", high = "#212121") +
  facet_grid(clade_name ~ root, scales = "free", space = "free") +
  xlab("COGs") +
  ylab("Species") +
  theme(
     legend.position    = "none"
    ,strip.background   = element_blank()
    ,strip.text.x       = element_text(size = 4, angle = 0, vjust = 0)
    ,strip.text.y       = element_text(size = 5, angle = 0, hjust = 0)
    ,axis.text.x        = element_text(size = 3, angle = -45, vjust = 0, hjust = 0.125)
    ,axis.text.y        = element_text(size = 4)
    ,axis.title         = element_text(size = 8)
    ,axis.ticks         = element_line(size = 0.25)
    ,panel.grid.major.y = element_line(size = 0.25)
    ,panel.grid.major.x = element_blank()
    ,panel.spacing      = unit(0.25, "pt")
    ,plot.margin        = unit(c(0,0,0,0), "mm")
  )
```

Supplementary Figure 2: Presence of orthologous groups in species. The horizontal axis is grouped according to assigned roots. The vertical axis is grouped by clades. A checkerboard pattern is superimposed to aid visualization.

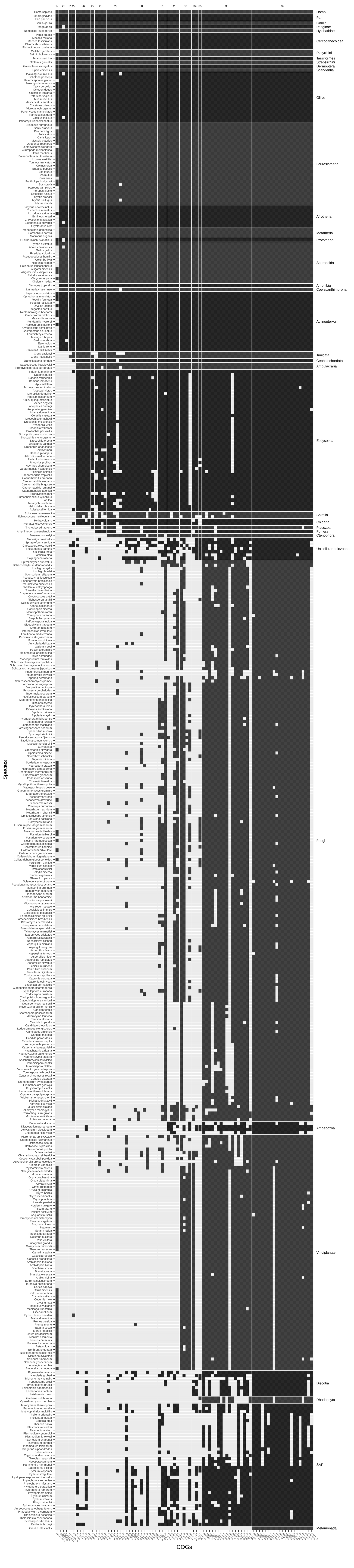

3.1.4 Adhesion genes

Aditionally, we also infer the roots of neural adhesion genes.

```
data(
  adhesion_genes,
  gene_cogs_extra,
  package = "neurotransmissionevolution"
)

cogs_of_interest_extra ← gene_cogs_extra %>% pull(cog_id) %>% unique

ogr_extra ← groot.preprocess(
  cogdata   = cogs,
  phyloTree = phyloTree,
  spid      = "9606",
  cogids    = cogs_of_interest_extra
)

roots_extra ← groot(ogr_extra, nPermutations = 1) %>%
  groot.get("results") %>%
  rownames_to_column("cog_id") %>%
  left_join(gene_cogs_extra) %>%
  left_join(adhesion_genes) %>%
  left_join(lca_names, by = c("Root" = "lca"))
```

Table 17: Adhesion genes in the neural system (KEGG Pathway hsa04514) and their roots.

String ID	COG	Symbol	Root
ENSP00000329797	NOG10515	CADM1	Human-Actinopterygii LCA (#20)
ENSP00000432943	NOG16799	MPZ	Human-Actinopterygii LCA (#20)
ENSP00000352513	NOG16799	MPZL1	Human-Actinopterygii LCA (#20)
ENSP00000361818	NOG22019	SDC4	Human-Actinopterygii LCA (#20)
ENSP00000357106	NOG09962	CADM3	Human-Tunicata LCA (#21)
ENSP00000264025	NOG09962	NECTIN1	Human-Tunicata LCA (#21)
ENSP00000418070	NOG09962	NECTIN3	Human-Tunicata LCA (#21)
ENSP00000370542	NOG05154	SDC1	Human-Ambulacraria LCA (#23)
ENSP00000307046	NOG05154	SDC2	Human-Ambulacraria LCA (#23)
ENSP00000344468	NOG05154	SDC3	Human-Ambulacraria LCA (#23)
ENSP00000265077	NOG02372	VCAN	Human-Cnidaria LCA (#26)
ENSP00000264638	KOG3516	CNTNAP1	Human-Placozoa LCA (#27)
ENSP00000354778	KOG3516	CNTNAP2	Human-Placozoa LCA (#27)
ENSP00000359085	KOG3512	NTNG1	Human-Placozoa LCA (#27)
ENSP00000376921	KOG3512	NTNG2	Human-Placozoa LCA (#27)
ENSP00000447006	KOG3513	CNTN1	Human-Ctenophora LCA (#29)
ENSP00000330633	KOG3513	CNTN2	Human-Ctenophora LCA (#29)
ENSP00000359077	KOG3513	L1CAM	Human-Ctenophora LCA (#29)
ENSP00000344786	KOG3513	NFASC	Human-Ctenophora LCA (#29)
ENSP00000368314	KOG3513	NRCAM	Human-Ctenophora LCA (#29)
ENSP00000261769	KOG3594	CDH1	Human-Unicellular holozoans LCA (#30)
ENSP00000269141	KOG3594	CDH2	Human-Unicellular holozoans LCA (#30)
ENSP00000379350	KOG1226	ITGB1	Human-Unicellular holozoans LCA (#30)
ENSP00000222573	KOG1226	ITGB8	Human-Unicellular holozoans LCA (#30)
ENSP00000480132	KOG3510	NCAM1	Human-Unicellular holozoans LCA (#30)
ENSP00000383392	KOG3510	NCAM2	Human-Unicellular holozoans LCA (#30)
ENSP00000350364	KOG3510	NEGR1	Human-Unicellular holozoans LCA (#30)
ENSP00000385142	KOG3514	NRXN1	Human-Unicellular holozoans LCA (#30)
ENSP00000265459	KOG3514	NRXN2	Human-Unicellular holozoans LCA (#30)
ENSP00000451648	KOG3514	NRXN3	Human-Unicellular holozoans LCA (#30)
ENSP00000367316	KOG3637	ITGA8	Human-Amoebozoa LCA (#32)
ENSP00000261023	KOG3637	ITGAV	Human-Amoebozoa LCA (#32)
ENSP00000376048	KOG4475	MAG	Human-Discoba LCA (#34)

ENSP00000392500	COG2272	NLGN1	Human-SAR LCA (#36)
ENSP00000305288	COG2272	NLGN2	Human-SAR LCA (#36)
ENSP00000351591	COG2272	NLGN3	Human-SAR LCA (#36)
ENSP00000370485	COG2272	NLGN4X	Human-SAR LCA (#36)
ENSP00000249363	COG4886	LRRC4	Human-Metamonada LCA (#37)
ENSP00000471502	COG4886	LRRC4B	Human-Metamonada LCA (#37)
ENSP00000278198	COG4886	LRRC4C	Human-Metamonada LCA (#37)
ENSP00000353030	COG5599	PTPRF	Human-Metamonada LCA (#37)
ENSP00000463325	COG5599	PTPRM	Human-Metamonada LCA (#37)

3.2 Neuroexclusivity

We characterize the relevance of a gene to the nervous system by inspecting what proportion of its activity is related to nervous processes. To do this, we rely on tissue RNA-Seq data, as well as KEGG's pathways themselves. Loading resources:

```
library(tidyverse)
library(magrittr)

data(
   gene_ids
  ,gene_pathways
  ,gene_expression
  ,package = "neurotransmissionevolution"
)
```

3.2.1 Expression neuroexclusivity

We start by averaging all `gene_expression` collected from the Expression Atlas by tissue (`tpm_avg`). The averaged expression is filtered for values greather than 0.5 TPM. This ensures further computations only account for tissues in which genes are actually expressed. Then, we add the manual tissue classification indicating which tissues are nervous or not (described in Preprocessing). The neuroexclusivity index of a gene is the sum of its `tpm_avg` values in nervous tissues divided by the sum its values in all tissues.

```
tissue_classification <- read_tsv(
  file      = "../data/neuroexclusivity_classification_tissue.tsv"
 ,col_types = "ci"
)

# Averaging TPM expression by tissue
avg_by_tissue <- gene_expression %>%
  group_by(ensembl_id, tissue) %>%
  summarise(tpm_avg = mean(tpm)) %>%
  filter(tpm_avg >= 0.5) %>%
  left_join(tissue_classification)

# Measuring expression neuroexclusivity
expression_neuroexclusivity <- avg_by_tissue %>%
  group_by(ensembl_id) %>%
  summarise(expression_neuroexclusivity = sum(tpm_avg[is_nervous == 1])/sum(tpm_avg)) %>%
  write_tsv("neuroexclusivity_expression.tsv")
```

3.2.2 Pathway neuroexclusivity

To find the pathway neuroexclusivity of a gene, we simply divide the number of nervous pathways by the number of all pathways it participates in.

```
pathway_classification <- read_tsv(
   file      = "../data/neuroexclusivity_classification_pathway.tsv"
```

```
  ,col_types = "cci"
)

link_pathway_entrez <- read_tsv(
   file      = "../data-raw/download/link_pathway_entrez.tsv"
  ,col_names = c("entrez_id", "pathway_id")
  ,col_types = "cc"
)

# Removing "hsa:" prefix
link_pathway_entrez[["entrez_id"]] %<>% str_split_n("\\:", 2)

# Pathway data related to our genes of interest
selected_genes_pathways <- link_pathway_entrez %>%
  filter(entrez_id %in% gene_ids[["entrez_id"]]) %>%
  left_join(pathway_classification) %>%
  drop_na # Dropping general pathways

# Measuring pathway neuroexclusivity
pathway_neuroexclusivity <- selected_genes_pathways %>%
  group_by(entrez_id) %>%
  summarise(pathway_neuroexclusivity = sum(is_nervous)/length(is_nervous)) %>%
  write_tsv("neuroexclusivity_pathway.tsv")
```

3.3 Network

This section describes the steps for reproducing most of the manuscript figures. A lot of it is concerned with plotting particularities and visual styling.

3.3.1 Graph data

Loading resources:

```
# Data manipulation
library(tidyverse)
library(igraph)
library(magrittr)
library(fuzzyjoin)

# Plotting dependencies
library(scatterpie)
library(UpSetR)
library(gridExtra)
library(patchwork)

# Utils
library(neurotransmissionevolution)

# Packaged data
data(
   gene_ids
  ,gene_cogs
  ,gene_pathways
  ,string_edgelist
  ,package = "neurotransmissionevolution"
)

# Fresh analysis data
cog_roots                   <- read_tsv("geneplast_roots.tsv",             col_types = "ci")
clade_names                 <- read_tsv("geneplast_clade_names.tsv",       col_types = "ic")
pathway_neuroexclusivity    <- read_tsv("neuroexclusivity_pathway.tsv",    col_types = "cn")
expression_neuroexclusivity <- read_tsv("neuroexclusivity_expression.tsv", col_types = "cn")

# Collapsing similar functions
gene_annotation <- read_tsv("../data/gene_annotation.tsv", col_types = "cc") %>%
  mutate(annotation = case_when(
     grepl("clearance",   annotation) ~ "depletion"
    ,grepl("degradation", annotation) ~ "depletion"
    ,grepl("transport",   annotation) ~ "synthesis"
    ,TRUE ~ annotation
  ))
```

We start by joining all gene data and creating the graph object.

```r
# If a gene has more than 1 COG, select the most recent one.
gene_cogs %<>%
  inner_join(cog_roots) %>%
  group_by(string_id) %>%
  filter(root == min(root)) %>%
  inner_join(clade_names)

# Gathering all gene info available
vertices <- gene_ids %>%
  na.omit %>%
  inner_join(gene_cogs) %>%
  inner_join(gene_pathways) %>%
  inner_join(gene_annotation) %>%
  inner_join(pathway_neuroexclusivity) %>%
  inner_join(expression_neuroexclusivity) %>%
  mutate(ne = pathway_neuroexclusivity >= 0.9) %>%
  select(string_id, everything())

# Quick color hack to aid visualization
vertices %<>%
  unite(color, glutamatergic:dopaminergic, remove = F) %>%
  mutate(color = rainbow(color %>% n_distinct)[color %>% as.factor])

g <- graph_from_data_frame(string_edgelist, directed = F, vertices = vertices)

# Setting node sizes
V(g)$size <- V(g)$system_count %>% sqrt %>% multiply_by(5)
```

The following block calls an utility function that handles the force directed layout with the aid of a shiny web server and the VivaGraphJS javascript library. A computed layout is already available in this folder.

```r
if(file.exists("network_layout.tsv")) {
  layout <- read_tsv("network_layout.tsv", col_types = "dd") %>% as.matrix
} else {
  layout <- vivagraph(g, precompute_multiplier = 200, precompute_niter = 1000)
}

# inserting layout coordinates into graph object
V(g)$x <-  layout[, 1]
# layout matrix comes vertically flipped
V(g)$y <- -layout[, 2]
```

We use base ggplot2 to draw the network. Edges are represented by a common `geom_path` layer. The following block retrieves tidy edge coordinates for the `geom_path` calls. We also perform some data wrangling necessary for faceting the network plots according to the roots of genes.

```r
# Recreating the vertices data.frame, now with layout coordinates (lazy way)
vertices <- g %>%
  igraph::as_data_frame(what = "vertices") %>%
  rename(string_id = name) %>%
  mutate(pie_border = NA)

# The edges data.frames will be used to draw lines with geom_path
edges_scaffold <- string_edgelist %$%
  data.frame(
     string_id        = c(stringId_A, stringId_B)
    ,group            = 1:nrow(.)
    ,stringsAsFactors = FALSE
  )

edges <- edges_scaffold %>%
  # Joining vertices to obtain coordinates
  inner_join(vertices, by = "string_id") %>%

  # Determining from which root onwards an edge can be drawn
  group_by(group) %>%
  mutate(edge_root = min(root))

# Reference data.frame for inequality join
subsequent_duplicator <- vertices %>%
  distinct(root) %>%
  inner_join(clade_names) %>%
  transmute(
     facet_root = root
    ,root_name  = paste0("Human-", clade_name, " LCA (#", root,")") %>% fct_reorder(-root)
```

```r
  )

# Simply duplicating emerging edges in subsequent roots
edges_in_roots ← edges %>%
  fuzzy_inner_join(subsequent_duplicator, by = c("edge_root" = "facet_root"), match_fun = list(`⩾`)) %>%
  mutate(past = edge_root ≠ facet_root)

# Simply duplicating emerging nodes in subsequent roots
nodes_in_roots ← vertices %>%
  fuzzy_inner_join(subsequent_duplicator, by = c("root" = "facet_root"), match_fun = list(`⩾`)) %>%
  mutate(
     past        = root ≠ facet_root
    ,node_fill   = ifelse(past, "past", annotation)
    ,node_border = ifelse(!past & ne, "ne_border", node_fill)
    ,pie_border  = ifelse(past, "past", ifelse(ne, "ne_border", "no_border"))
  )
```

Setting up reusable aesthetic parameters to avoid code duplication.

```r
systems ← c(
   "cholinergic"
  ,"dopaminergic"
  ,"gabaergic"
  ,"glutamatergic"
  ,"serotonergic"
)

edge_color ← rgb(0.7, 0.7, 0.7, alpha = 0.3)

color_mappings ← c(
  #----- neurotransmitter systems ----
   "cholinergic"           = "#D84315"
  ,"dopaminergic"          = "#F9A825"
  ,"gabaergic"             = "#558B2F"
  ,"glutamatergic"         = "#1565C0"
  ,"serotonergic"          = "#6A1B9A"
  #--- neurotransmission functions ---
  ,"depletion"             = "#F40000"
  ,"excitability"          = "#FFAB00"
  ,"receptor-associated"   = "#D6EE00"
  ,"ionotropic receptor"   = "#43FF1C"
  ,"metabotropic receptor" = "#18FFFF"
  ,"signaling"             = "#0091EA"
  ,"g-protein"             = "#0033ff"
  ,"synthesis"             = "#AA00FF"
  ,"vesicle"               = "#FF00AA"
  #------- is_neuroexclusive ---------
  ,"TRUE"                  = "#00BFC4"
  ,"FALSE"                 = "#F8766D"
  #-------- network faceting ---------
  ,"past"                  = "#FFFFFF"
  ,"other"                 = "#A0A0A0"
  ,"other_edge"            = "#B3B3B3"
  ,"ne_border"             = "#000000"
)

# Some recurrent ggplot aesthetics
edge_aes ← aes(x = x, y = y, group = group)
pie_aes  ← aes(x = x, y = y, color = pie_border, group = string_id, r = size^(0.94) - 1.5)

theme_facet ← list(
   # Scales
   scale_fill_manual(values = color_mappings)
  ,scale_color_manual(values = color_mappings %>% darken(0.25) %>% c("no_border" = NA), guide = FALSE)
  ,scale_shape_manual(values = c("TRUE" = "square filled", "FALSE" = "circle filled"), guide = FALSE)
  ,coord_equal()
  # Theme
  ,theme_void()
  ,theme(
     strip.text       = element_text(margin = margin(10, 10, 10, 10))
    ,strip.text.x     = element_text(color = "#616161", size = 10)
    ,strip.background = element_rect(color = "#E0E0E0", fill = "#E0E0E0", size = 0.5, linetype = "solid")
    ,panel.border     = element_rect(color = "#E0E0E0", fill = NA, size = 0.5, linetype = "solid")
  )
  ,guides(legend.position="none")
  ,guides(fill = FALSE, color = FALSE, linetype = FALSE, shape = FALSE, size = FALSE)
)
```

3.3.2 Manuscript figure 1

```r
# Common elements
plot_edges <- geom_path(
    data    = edges
  ,mapping = edge_aes
  ,color   = edge_color
  ,size    = 0.1
)

plot_text <- geom_text(
    data    = vertices
  ,mapping = aes(x, y, label = string_name)
  ,size    = 1.5
  ,vjust   = 0
  ,nudge_y = 6
  ,alpha   = 0.5
)

# Figure 1A
plot_pies <- geom_scatterpie(
    data    = vertices
  ,mapping = pie_aes
  ,cols    = systems
)

fig1a <- ggplot() +
  plot_edges +
  plot_pies +
  theme_facet +
  plot_text +
  guides(fill="legend")

# Figure 1B
plot_nodes <- geom_point(
    data    = vertices
  ,mapping = aes(x, y, fill = annotation, color = annotation, size = size)
  ,shape   = 21
  ,stroke  = 1
)

fig1b <- ggplot() +
  plot_edges +
  plot_nodes +
  theme_facet +
  plot_text +
  scale_radius(range = c(1.5, 4.5), guide = FALSE) +
  guides(fill="legend")

# Plotting and saving
fig1a / fig1b
```

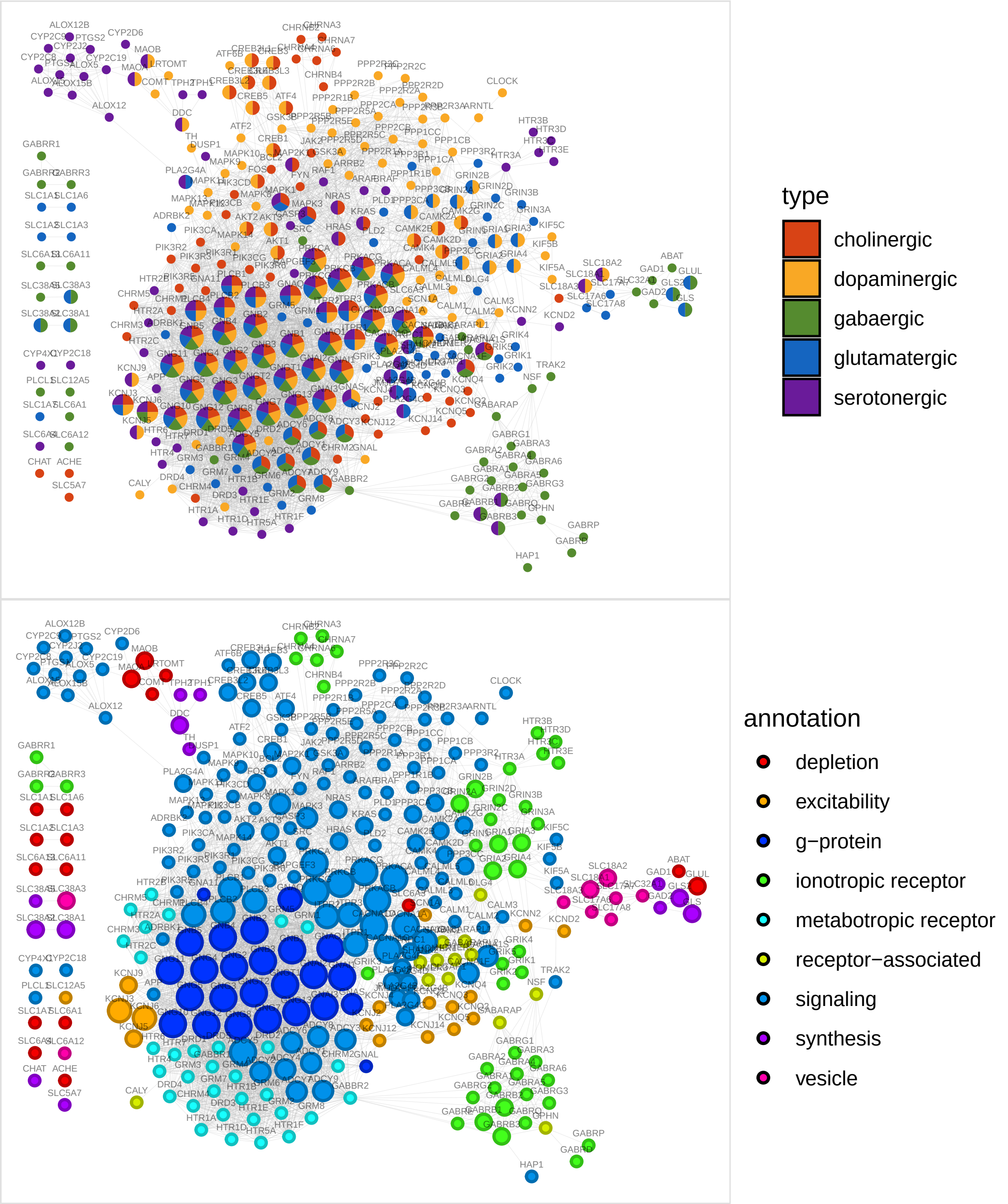

Supplementary Figure 3: Unedited manuscript figure 1. The human neurotransmission network with nodes colored by neurotransmitter systems (top) and neurotransmission functions (bottom).

3.3.3 Manuscript figure 2

This figure is produced externally by a program called ViaComplex, which superimposes a heatmap over the network layout based on a node property. In our case, this property is the neuroexclusivity index. The following block handles data formatting related to ViaComplex.

```
# Retrieving the largest connected component
subgraphs <- decompose.graph(g)
lcc_index <- which.max(sapply(subgraphs, vcount))
lcc       <- subgraphs[[lcc_index]]

# Writing network data to viacomplex's custom format (similar to pajek)
# xy_hack adds some extra margin to the plot
xy_hack <- data.frame(
   name                          = c("top", "bot")
  ,x                             = range(V(lcc)$x) + c(-75, 75)
  ,y                             = range(V(lcc)$y) + c(-75, 75)
  ,pathway_neuroexclusivity    = 0
  ,expression_neuroexclusivity = 0
  ,stringsAsFactors            = F
)

pajek_nodes <- lcc %>%
  igraph::as_data_frame("vertices") %>%
  bind_rows(xy_hack) %>%
  mutate(id = row_number(), y = -y)

pajek_edges <- igraph::as_data_frame(lcc, "edges")

# Creating the network_viacomplex.net file and sequentially populating it
write("*edges", "network_viacomplex.net")
write_tsv(
   x            = pajek_edges
  ,path         = "network_viacomplex.net"
  ,append       = T
  ,col_names    = F
  ,quote_escape = F
)
write("*nodes", "network_viacomplex.net", append = T)
write_tsv(
   x            = pajek_nodes %>% select(name, x, y)
  ,path         = "network_viacomplex.net"
  ,append       = T
  ,col_names    = F
  ,quote_escape = F
)

write_tsv(
   x    = pajek_nodes %>% select(id, name, pathway_neuroexclusivity)
  ,path = "network_viacomplex_pathway.dat"
)
write_tsv(
   x    = pajek_nodes %>% select(id, name, expression_neuroexclusivity)
  ,path = "network_viacomplex_expression.dat"
)
```

3.3.4 Manuscript figure 3

The process for generating Figures 3 and 4 is roughly the same. It consists of finding what nodes have numeric roots in a given range. In our analysis, the largest root is numbered 37 and represents the oldest common ancestor to humans in the cladogram (the Human-Metamonada LCA, as seen in previous sections). Root number 1 is represented by *Homo sapiens* itself.

```
# Subsetting node data for the two roots
nodes_in_roots_fig3 <- nodes_in_roots %>%
  filter(facet_root %in% c(37,26)) %>%
  mutate(
     # Correctly color coding every node between these roots
     past        = (root == 37) & (root != facet_root)
    ,pie_border  = ifelse(past, "past", ifelse(ne, "ne_border", "no_border"))
  )

# Nodes from root 37 should be white in root 26
```

```r
nodes_in_roots_fig3[nodes_in_roots_fig3[["past"]], systems] <- 0

ggplot() +
  # Edges
  geom_path(
     data     = edges_in_roots %>% filter(facet_root %in% c(37,26))
    ,mapping = edge_aes
    ,color   = edge_color
    ,size    = 0.1
  ) +
  # Non-neuroexclusive nodes
  geom_scatterpie(
     data     = nodes_in_roots_fig3 %>% filter(ne == FALSE)
    ,mapping = pie_aes
    ,cols    = systems %>% c("past")
  ) +
  # Neuroexclusive nodes
  geom_scatterpie(
     data     = nodes_in_roots_fig3 %>% filter(ne == TRUE)
    ,mapping = pie_aes
    ,cols    = systems %>% c("past")
    ,n       = 5 # Square nodes
  ) +
  geom_text(
     data     = nodes_in_roots_fig3 %>% filter(past == FALSE)
    ,mapping = aes(x = x, y = y + linMap(system_count, 10, 16), label = string_name)
    ,size    = 1.5
    ,alpha   = 0.5
  ) +
  facet_wrap(. ~ root_name, ncol = 1) +
  theme_facet
```

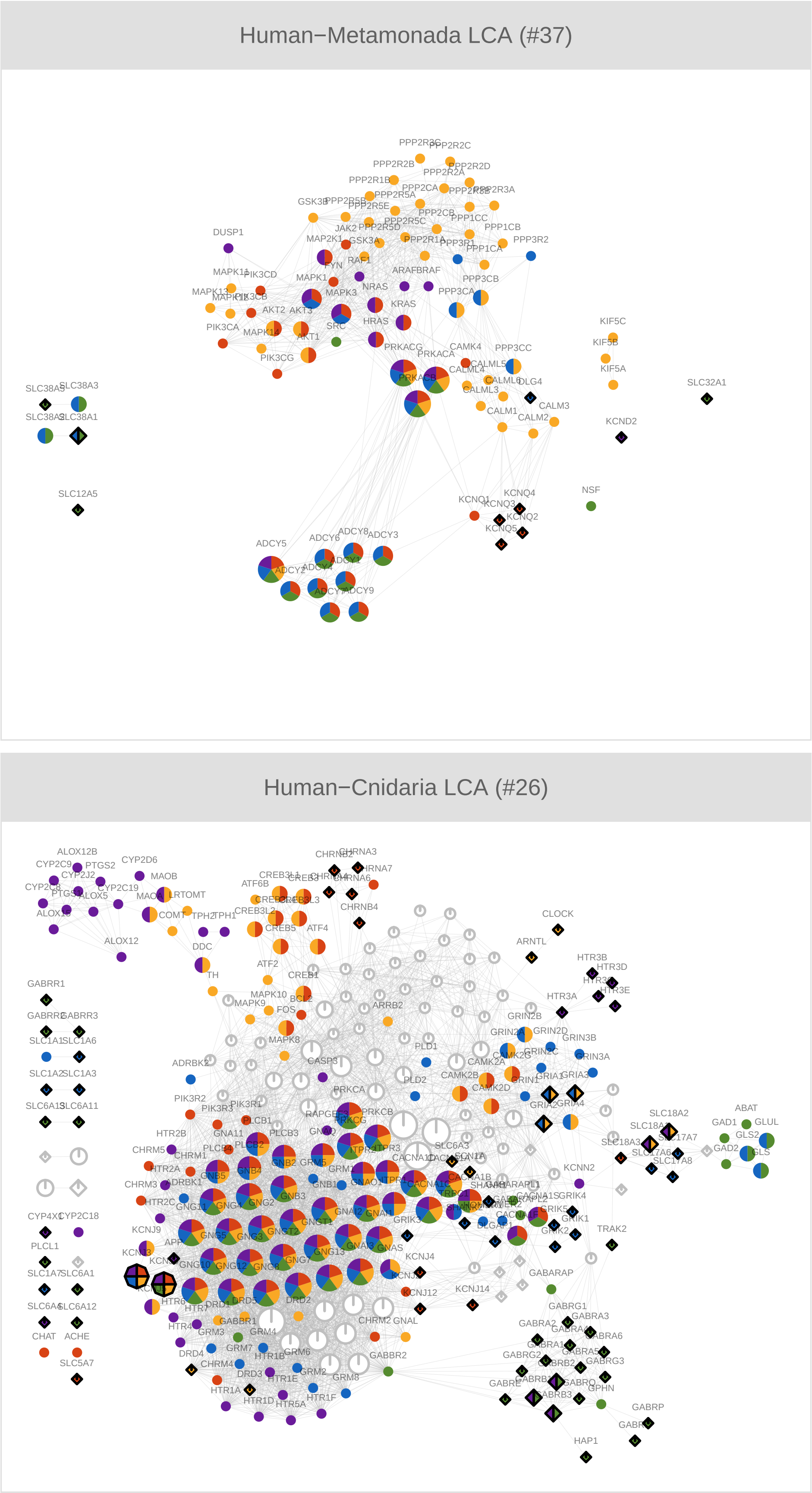

Supplementary Figure 4: Unedited manuscript figure 3. It represents the human neurotransmission network with nodes colored by neurotransmitter systems. At the top, we have the human network composed only of nodes rooted at the origin of Eukaryota. At the bottom, we have the human network composed of nodes rooted since the origin of Eukaryota until the Human-Cnidaria LCA.

Additionally, we cumulatively count nodes by their categories (function and neuroexclusivity) and inferred root:

```r
cumulative_emergence <- vertices %>%
  select(root, annotation, is_neuroexclusive = ne) %>%
  # Adding clade info
  right_join(clade_names) %>%
  # Converting logical to character
  mutate(is_neuroexclusive = as.character(is_neuroexclusive)) %>%
  # Pivoting from wide to long
  pivot_longer(annotation:is_neuroexclusive, values_ptypes = list(value = "character")) %>%
  # Counting nodes by category (name) for each root
  count(root, clade_name, name, value) %>%
  # Making absent counts explicit
  group_by(name) %>%
  tidyr::complete(nesting(root, clade_name), name, value, fill = list(n = 0)) %>%
  # No reason to include NA observations in cumulative sum
  na.omit %>%
  # Cumulative sum node count at each root
  group_by(name, value) %>%
  mutate(cumulative_count = order_by(-root, cumsum(n)))
```

Plotting such cumulative counts:

```r
# Creating ordered factors for plotting
cumulative_emergence %<>% ungroup %>%
  mutate(
     clade_name = fct_reorder(clade_name, -root)
    ,value      = fct_reorder(value, name)
  )

ggplot(cumulative_emergence) +
  # Barplot
  geom_bar(
     mapping     = aes(clade_name, cumulative_count, group = value)
    ,stat        = "sum"
    ,fill        = "#999999"
    ,show.legend = F
  ) +
  # Lines
  geom_line(
     mapping = aes(clade_name, cumulative_count, group = value, color = value)
    ,size    = 1
  ) +
  # Styling
  scale_color_manual(values = color_mappings) +
  facet_grid(name ~ .) +
  theme(
     axis.title  = element_blank()
    ,axis.text.x = element_text(size = 12, angle = -45, vjust = 0, hjust = 0)
  )
```

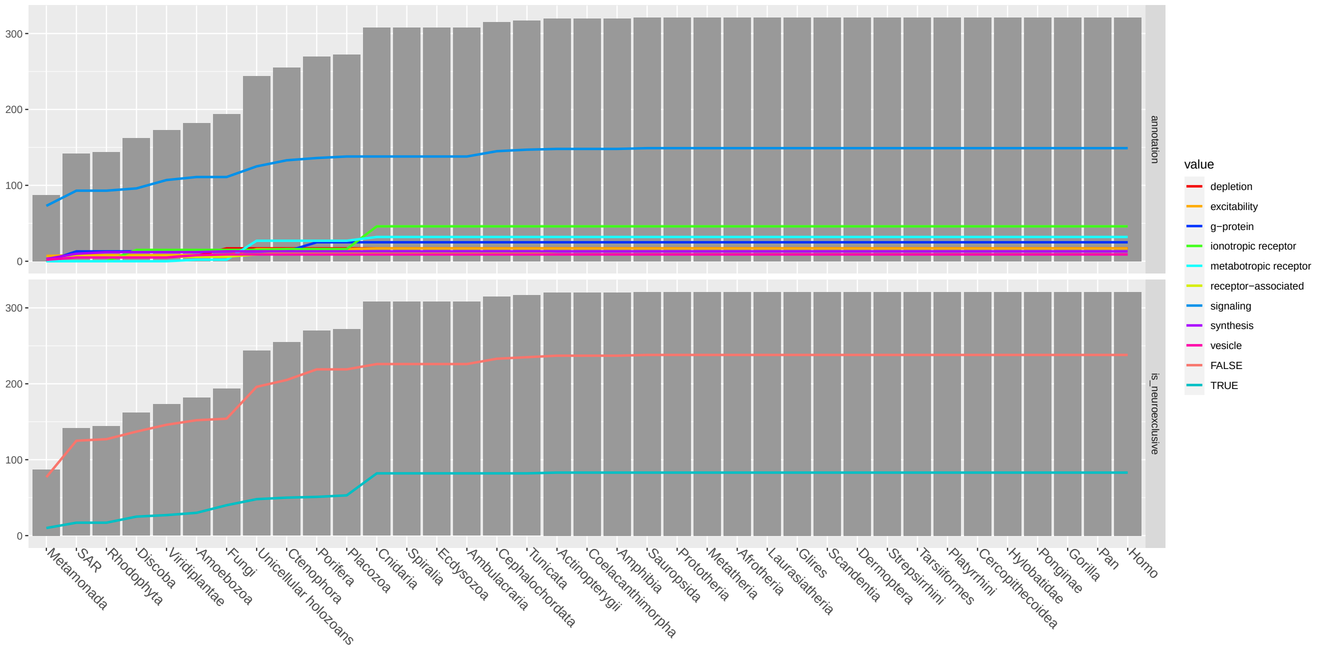

Supplementary Figure 5: Cumulative node counts by categories at each root.

3.3.5 Manuscript figure 4

Visualizing nodes with roots <= 30 (Human-Porifera LCA) and >= 26 (Human-Cnidaria LCA) at every distinct root.

```
ggplot() +
  # Edges
  geom_path(
     data     = edges_in_roots %>% filter(facet_root >= 26 & facet_root <= 30)
    ,mapping  = edge_aes
    ,color    = edge_color
    ,size     = 0.1
  ) +
  # Nodes
  geom_point(
     data = nodes_in_roots %>% filter(facet_root >= 26 & facet_root <= 30)
    ,mapping = aes(
       x      = x
      ,y      = y
      ,size   = size
      ,shape = ne
      ,fill  = node_fill
      ,color = node_border
    )
  ) +
  # Labels
  geom_text(
     data = nodes_in_roots %>% filter(past == FALSE & facet_root >= 26 & facet_root <= 30)
    ,mapping = aes(x = x, y = y + linMap(system_count, 10, 16), label = string_name)
    ,size = 2
    ,alpha = 0.75
  ) +
  scale_radius(range = c(1.75, 4.5)) +
  facet_wrap(. ~ root_name, ncol = 5) +
  theme_facet
```

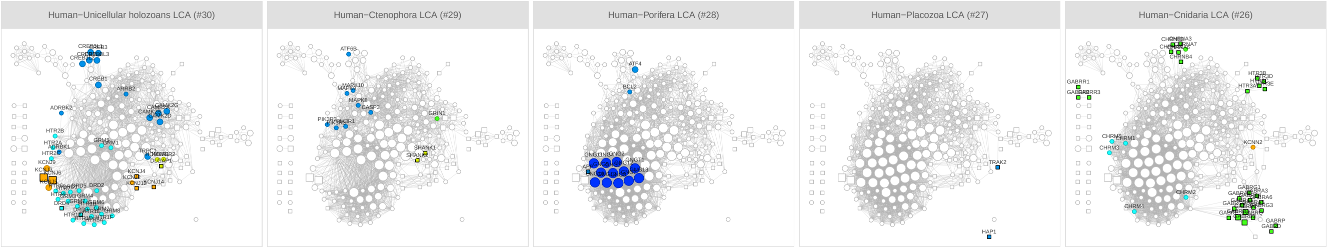

Supplementary Figure 6: Unedited manuscript figure 4. The human neurotransmission network composed of nodes rooted between roots 30 (Holozoa LCA) and 26 (Human-Cnidaria LCA).

3.3.6 Supplementary network figures

The following supplementary figures help us see what nodes have been rooted at each LCA. Nodes from previous roots are colored white.

```
ggplot() +
  # Edges
  geom_path(
     data    = edges_in_roots
    ,mapping = edge_aes
    ,color   = edge_color
    ,size    = 0.1
  ) +
  # Nodes
  geom_point(
     data = nodes_in_roots
    ,mapping = aes(
       x     = x
      ,y     = y
      ,size  = size
      ,shape = ne
      ,fill  = node_fill
      ,color = node_border
    )
  ) +
  # Labels
  geom_text(
     data = nodes_in_roots %>% filter(past == FALSE)
    ,mapping = aes(x = x, y = y + linMap(system_count, 10, 15), label = string_name)
    ,size = 1.15
    ,alpha = 0.5
  ) +
  scale_radius(range = c(1.5, 3.5)) +
  facet_wrap(. ~ root_name, ncol = 1) +
  theme_facet
```

Supplementary Figure 7: The human neurotransmission network with nodes rooted at each human LCA. Nodes are colored according to their function in neurotransmission. Nodes from previous roots are colored white. Nodes with black borders are neuroexclusive.

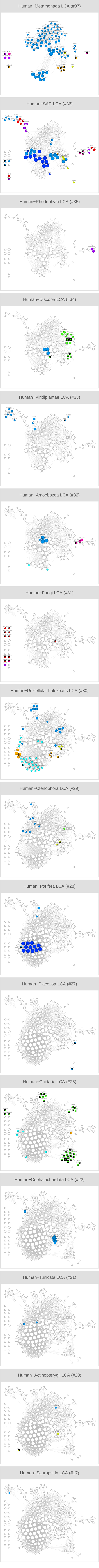

```r
# Past nodes should be white (ie have no systems)
nodes_in_roots[nodes_in_roots[["past"]], systems] <- 0

ggplot() +
  # Edges
  geom_path(
     data    = edges_in_roots
    ,mapping = edge_aes
    ,color   = edge_color
    ,size    = 0.1
  ) +
  # Non-neuroexclusive nodes
  geom_scatterpie(
     data    = nodes_in_roots %>% filter(ne == FALSE)
    ,mapping = pie_aes
    ,cols    = systems %>% c("past")
  ) +
  # Neuroexclusive nodes
  geom_scatterpie(
     data    = nodes_in_roots %>% filter(ne == TRUE)
    ,mapping = pie_aes
    ,cols    = systems %>% c("past")
    ,n       = 5 # Square nodes
  ) +
  # Labels
  geom_text(
     data = nodes_in_roots %>% filter(past == FALSE)
    ,mapping = aes(x = x, y = y + linMap(system_count, 10, 16), label = string_name)
    ,size = 2.25
    ,alpha = 0.5
  ) +
  facet_wrap(. ~ root_name, ncol = 1) +
  theme_facet +
  theme(
     strip.text       = element_text(margin = margin(20, 20, 20, 20))
    ,strip.text.x     = element_text(color = "#616161", size = 20)
    ,strip.background = element_rect(color = "#E0E0E0", fill = "#E0E0E0", size = 0.5, linetype = "solid")
    ,panel.border     = element_rect(color = "#E0E0E0", fill = NA, size = 0.5, linetype = "solid")
  )
```

Supplementary Figure 8: The human neurotransmission network with nodes rooted at each human LCA. Nodes are colored according to their neurotransmitter systems. Nodes from previous roots are colored white. Nodes with black borders are neuroexclusive.

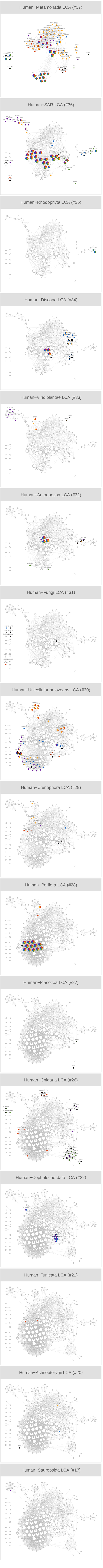

```r
# Pivoting vertices data.frame to enable faceting by systems
vertices_long <- pivot_longer(
   data      = vertices
  ,cols      = all_of(systems)
  ,names_to  = "system"
  ,values_to = "is_system"
) %>%
  mutate(
     system_color     = ifelse(is_system,     system, "other")
    ,annotation_color = ifelse(is_system, annotation, "other")
  )

edges_systems <- edges_scaffold %>%
  inner_join(vertices_long, by = "string_id") %>%
  # Determines from which root onwards and edge can be drawn (as well as its color)
  group_by(group, system) %>%
  mutate(
     edge_system = ifelse(is_system[which.min(root)], system, "other_edge")
    ,edge_root   = min(root)
  )

# Simply duplicating emerging edges in subsequent roots
edges_in_roots <- edges_systems %>%
  fuzzy_inner_join(subsequent_duplicator, by = c("edge_root" = "facet_root"), match_fun = list(`>=`)) %>%
  mutate(
     past = edge_root != facet_root
    ,edge_color = ifelse(!past & (system_color == edge_system), system, "other_edge")
  )

# Simply duplicating emerging nodes in subsequent roots
nodes_in_roots <- vertices_long %>%
  fuzzy_inner_join(subsequent_duplicator, by = c("root" = "facet_root"), match_fun = list(`>=`)) %>%
  mutate(
     past              = root != facet_root
    ,system_color      = ifelse(past, "past", system_color)
    ,system_border     = ifelse(!past & ne, "ne_border", system_color)
    ,annotation_border = ifelse(!past & ne, "ne_border", annotation_color)
  )

ggplot() +
  # Edges
  geom_path(
     data    = edges_in_roots
    ,mapping = edge_aes
    ,color   = color_mappings[edges_in_roots[["edge_color"]]] %>% alpha(0.3)
    ,size    = 0.1
  ) +
  # Nodes
  geom_point(
     data = nodes_in_roots
    ,mapping = aes(
       x     = x
      ,y     = y
      ,size  = size
      ,shape = ne
      ,fill  = system_color
      ,color = system_border
    )
  ) +
  # Labels
  geom_text(
     data = nodes_in_roots %>% filter(past == FALSE & is_system == TRUE)
    ,mapping = aes(x = x, y = y + linMap(system_count, 10, 16), label = string_name)
    ,size = 1.5
    ,alpha = 0.75
  ) +
  scale_radius(range = c(1, 3)) +
  facet_grid(root_name ~ system, switch = "y") +
  theme_facet
```

Supplementary Figure 9: The human neurotransmission network with nodes rooted at each human LCA. Nodes are colored according to their neurotransmitter systems. Nodes from previous roots are colored white. Nodes with black borders are neuroexclusive.

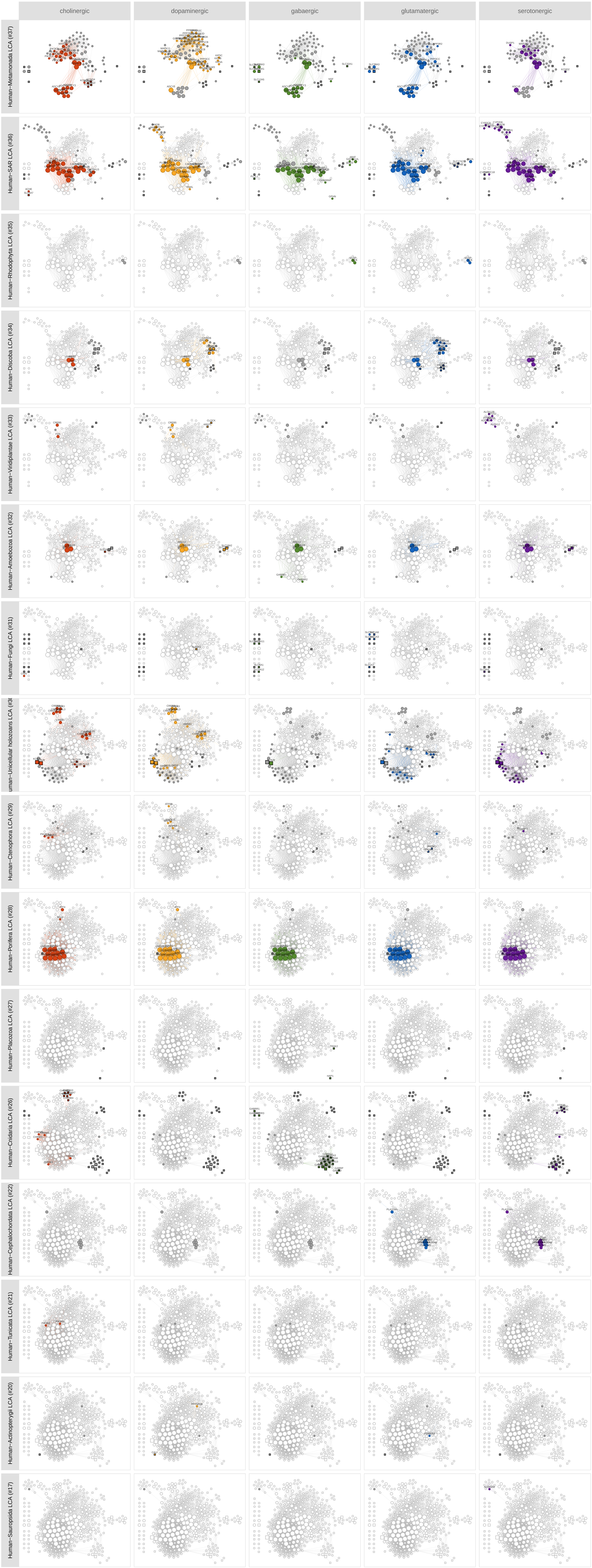

3.3.7 Manuscript set diagrams

The following code block produces all set diagrams from the main manuscript.

```r
upset_texts <- c(
   2   #ytitle
  ,2   #ytick
  ,1   #setsizetitle
  ,1.5 #setsizetick
  ,2   #setnames
  ,2.5 #barnums
)

# We have to manually find the correct order of colors
# Because UpSetR does not understand named vectors
get_colors <- function(df) {
  ordered_systems <- df %>%
    select(systems) %>%
    colSums %>%
    extract(. > 0) %>%
    extract(order(., names(.), decreasing = T))

  color_mappings[names(ordered_systems)]
}

# Figure 1A set diagram
upset(
   select(vertices, systems)
  ,mb.ratio        = c(0.7, 0.3)
  ,order.by        = "freq"
  ,mainbar.y.label = "System Intersections"
  ,sets.x.label    = "Genes per system"
  ,text.scale      = upset_texts
  ,point.size      = 3.5
  ,line.size       = 1
  ,sets.bar.color  = get_colors(vertices)
)
dev.print(pdf, "plots/fig1a_set_raw.pdf", width = 18, height = 10, onefile = F, useDingbats = F)

# Figure 3A set diagram
fig3a_set <- vertices %>% filter(root == 37) %>% select(systems)
upset(
   fig3a_set
  ,mb.ratio        = c(0.7, 0.3)
  ,order.by        = "freq"
  ,mainbar.y.label = "System Intersections"
  ,sets.x.label    = "Genes per system"
  ,text.scale      = upset_texts
  ,point.size      = 3.5
  ,line.size       = 1
  ,sets.bar.color  = get_colors(fig3a_set)
)
dev.print(pdf, "plots/fig3a_set_raw.pdf", width = 16, height = 8, onefile = F, useDingbats = F)

# Figure 3B set diagram
fig3b_set <- vertices %>% filter(root < 37 & root >= 26) %>% select(systems)
upset(
   fig3b_set
  ,mb.ratio        = c(0.7, 0.3)
  ,order.by        = "freq"
  ,mainbar.y.label = "System Intersections"
  ,sets.x.label    = "Genes per system"
  ,text.scale      = upset_texts
  ,point.size      = 3.5
  ,line.size       = 1
  ,sets.bar.color  = get_colors(fig3b_set)
)
dev.print(pdf, "plots/fig3b_set_raw.pdf", width = 16, height = 8, onefile = F, useDingbats = F)
```

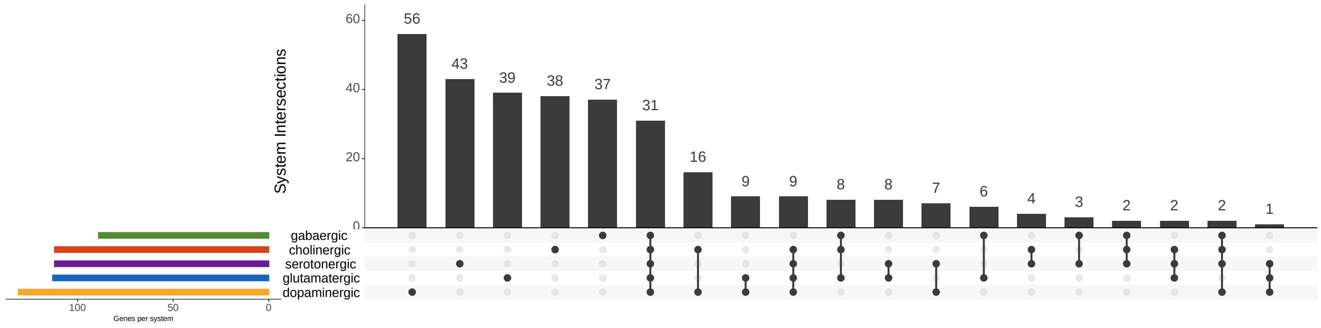

(a) Set diagram for Figure 1A

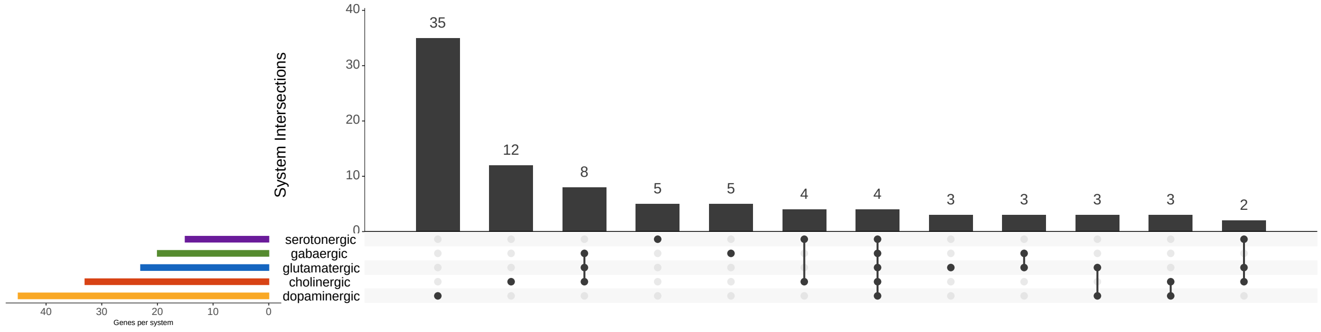

(b) Set diagram for Figure 3A

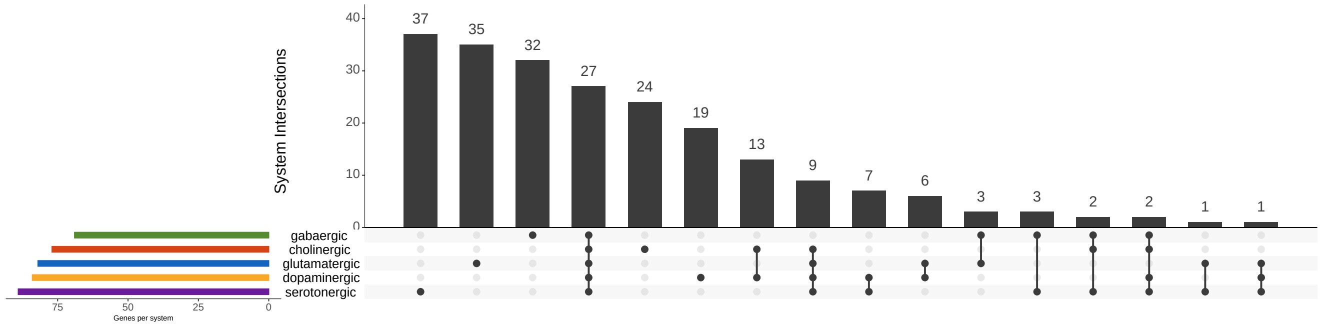

(c) Set diagram for Figure 3B

Supplementary Figure 10: Set diagrams

3.4 Abundance

Abundance is the number of proteins of a single species in a single orthologous group. Abundances are aggregated and averaged according to the function of their corresponding orthogroups. The function of an orthogroup is considered to be the function of its human proteins. Loading initial resources:

```
# Data manipulation
library(tidyverse)
library(magrittr)

# Utils
library(neurotransmissionevolution)

# Packaged data
data(
   cogs
  ,gene_ids
  ,gene_cogs
  ,string_eukaryotes
  ,package = "neurotransmissionevolution"
```

```
)

# Fresh analysis data
cog_roots                   <- read_tsv("geneplast_roots.tsv",             col_types = "ci")
clade_names                 <- read_tsv("geneplast_clade_names.tsv",       col_types = "ic")
clade_taxids                <- read_tsv("geneplast_clade_taxids.tsv",      col_types = "ici")

# Collapsing similar functions
gene_annotation <- read_tsv("../data/gene_annotation.tsv", col_types = "cc") %>%
  mutate(annotation = case_when(
     grepl("clearance",   annotation) ~ "depletion"
    ,grepl("degradation", annotation) ~ "depletion"
    ,grepl("transport",   annotation) ~ "synthesis"
    ,TRUE ~ annotation
  ))
```

We start by setting up reusable data frames with useful metrics.

```
# If a gene has more than 1 COG, select the most recent one.
gene_cogs %<>%
  inner_join(cog_roots) %>%
  group_by(string_id) %>%
  filter(root == min(root))

# The function of a COG is the function of its proteins
cog_annotation <- gene_ids %>%
  inner_join(gene_cogs) %>%
  inner_join(gene_annotation) %>%
  distinct(cog_id, annotation)

# Number of proteins in a COG in every species
cog_abundance_by_taxid <- cogs %>%
  filter(cog_id %in% gene_cogs[["cog_id"]]) %>%
  count(taxid, cog_id,  name = "abundance") %>%
  left_join(cog_annotation)

# Mapping species to clade info
ordered_species <- string_eukaryotes %>%
  select(taxid, ncbi_name) %>%
  left_join(clade_taxids) %>%
  left_join(clade_names, by = c("lca" = "root")) %>%
  mutate(
     ncbi_name  = fct_reorder(ncbi_name, -taxid_order)
    ,clade_name = fct_reorder(clade_name, -taxid_order)
  )

# Plotting colors
annotation_colors <- c(
   "depletion"             = "#F40000"
  ,"excitability"          = "#FFAB00"
  ,"receptor-associated"   = "#D6EE00"
  ,"ionotropic receptor"   = "#43FF1C"
  ,"metabotropic receptor" = "#18FFFF"
  ,"signaling"             = "#0091EA"
  ,"g-protein"             = "#0033ff"
  ,"synthesis"             = "#AA00FF"
  ,"vesicle"               = "#FF00AA"
)
```

The average orthogroup abundances are finally computed for each species according to the function of orthogroups.

```
avg_abundance_by_function <- cog_abundance_by_taxid %>%
  group_by(taxid, annotation) %>%
  summarise(avg_abundance = mean(abundance)) %>%
  # Adding species and clade info
  left_join(ordered_species)
```

Plotting:

```
# This vertical line indicates the first metazoan (Mnemiopsis leidyi / Ctenophora)
metazoa_line <- geom_vline(
   xintercept = "Mnemiopsis leidyi"
  ,color      = "#FF0000"
  ,linetype   = "11"
```

```r
  ,alpha      = 1
  ,size       = 0.25
)

# Facet labeller
annotation_labels <- c(
   "ionotropic receptor"   = "Ionotropic \nreceptor"
  ,"metabotropic receptor" = "Metabotropic \nreceptor"
  ,"receptor-associated"   = "Receptor-\nassociated"
  ,"signaling"             = "Signaling"
  ,"vesicle"               = "Vesicle"
  ,"synthesis"             = "Synthesis"
  ,"depletion"             = "Depletion"
  ,"g-protein"             = "G protein"
  ,"excitability"          = "Excitability"
)

# Custom tick function
tick_function <- function(x) {
  seq(x[2], 0, length.out = 3) %>% head(-1) %>% tail(-1) %>% { ceiling(./5)*5 }
}

# Capping abundance values based on metazoan mean
capped_abundance_by_function <- avg_abundance_by_function %>%
  # mutate(capped_abundance = ifelse(abundance >= 100, 100, abundance)) %>%
  group_by(annotation) %>%
  mutate(
     max_abundance = avg_abundance[lca <= 29] %>% { mean(.) + 3*sd(.) }
    ,avg_abundance = ifelse(avg_abundance >= max_abundance, pmin(max_abundance, 100), pmin(avg_abundance, 100))
  )

# Plotting
abundance_plot <- ggplot(capped_abundance_by_function) +
  # Geoms  ---------------
  metazoa_line +
  geom_bar(
     aes(x = ncbi_name, y = avg_abundance, fill = annotation, color = after_scale(darken(fill, 0.1)))
    ,stat = "identity"
  ) +
  # Labels  --------------
  xlab("Species") +
  ylab("Average protein abundance in orthologous groups") +
  # Scales ---------------
  scale_y_continuous(breaks = tick_function, minor_breaks = NULL) +
  scale_fill_manual(values = annotation_colors %>% darken(0.1)) +
  # Styling --------------
  facet_grid(
     annotation ~ clade_name
    ,scales    = "free"
    ,space     = "free"
    ,labeller = labeller(annotation = annotation_labels)
  ) +
  theme_classic() +
  theme_main
ggsave("plots/fig5_raw.pdf", abundance_plot, width = 18, height = 6)

# Uncapped abundances for supplementary text
abundance_plot %+% avg_abundance_by_function +
  facet_grid(
     clade_name ~ annotation
    ,scales    = "free"
    ,space     = "free"
    ,labeller = labeller(annotation = annotation_labels)
  ) +
  coord_flip() +
  theme_supplementary
```

Supplementary Figure 11: Average protein abundances in orthologous groups by species. Orthologous groups are faceted according to their functions.

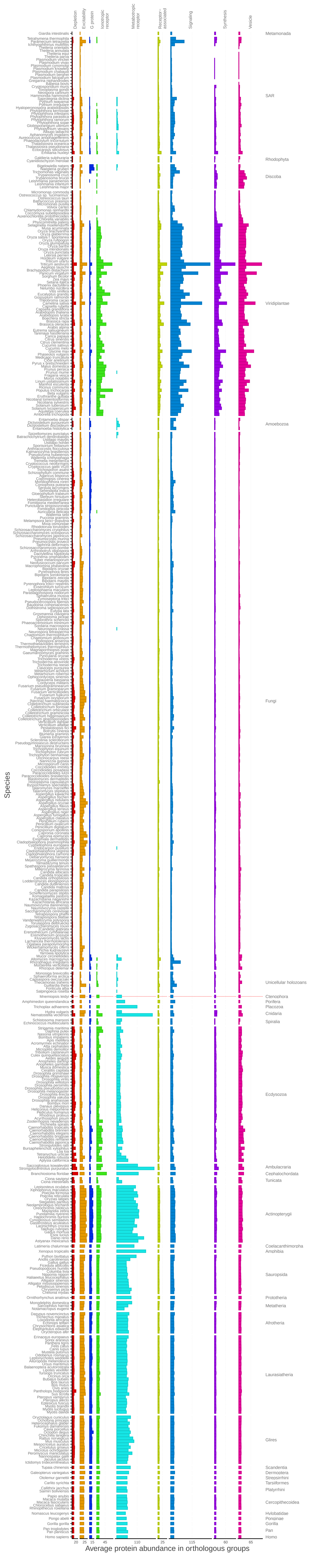

Species-specific average abundances, now averaged by clades:

```
ggplot(avg_abundance_by_function) +
  geom_bar(
    aes(x = clade_name, y = avg_abundance, fill = annotation, color = after_scale(darken(fill, 0.1)))
    ,stat = "summary"
    ,fun  = "mean"
  ) +
  scale_y_continuous(breaks = tick_function, minor_breaks = NULL) +
  scale_fill_manual(values = annotation_colors, guide = "none") +
  facet_grid(
     annotation ~ .
    ,scales    = "free"
    ,space     = "free_y"
    ,labeller = labeller(annotation = sub("\n", "", annotation_labels))
  ) +
  xlab("Clades") +
  ylab("Average abundance averaged by clade") +
  theme_classic() +
  theme_average
```

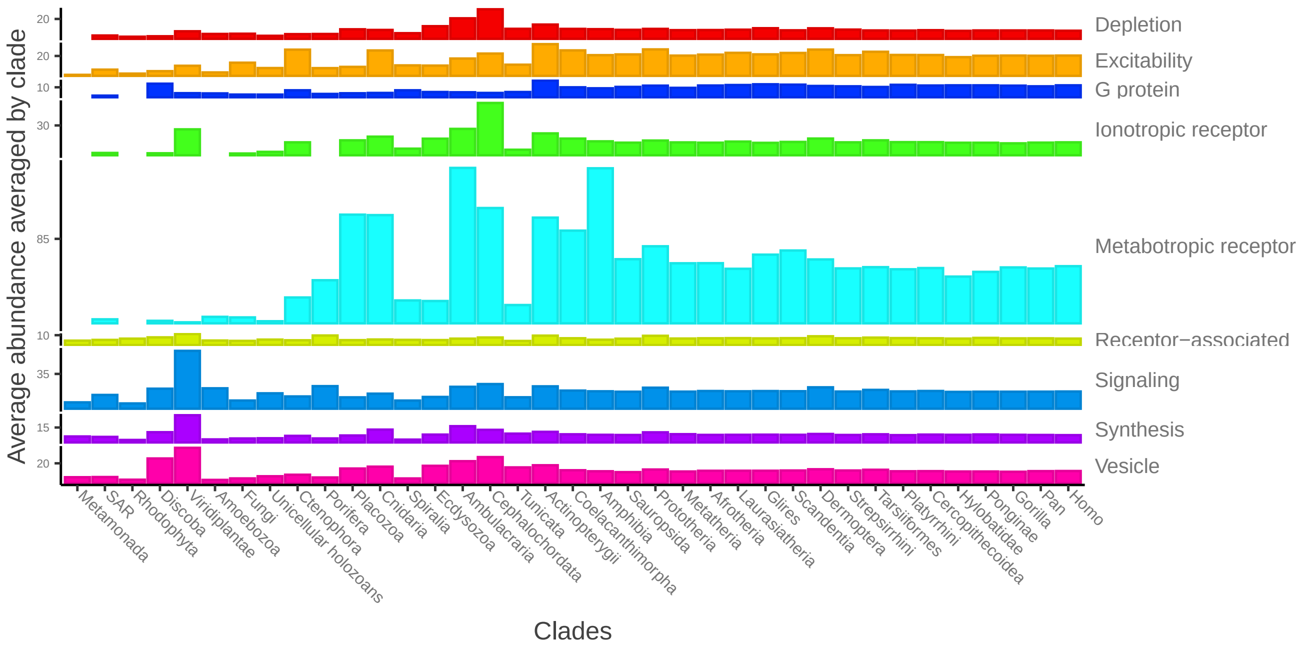

Supplementary Figure 12: Species-specific average abundances, now averaged by clades.

Plain protein abundance in single orthogroups:

```
# Collapsing COGs with multiple functions
cog_abundance_collapsed <- cog_abundance_by_taxid %>%
  group_by(taxid, cog_id) %>%
  summarise(
     annotation = paste(annotation, collapse = "/")
    ,abundance  = unique(abundance)
  ) %>%
  ungroup %>%
  left_join(ordered_species) %>%
  arrange(annotation) %>%
  mutate(cog_id = fct_inorder(cog_id))

# Adding colors for such COGs
annotation_colors %<>% c(
   "vesicle/synthesis"      = "#808080"
  ,"depletion/vesicle"      = "#808080"
  ,"signaling/excitability" = "#808080"
)
```

```r
ggplot(cog_abundance_collapsed) +
  metazoa_line +
  geom_bar(aes(x = ncbi_name, y = abundance, fill = annotation), stat = "identity") +
  scale_fill_manual(values = annotation_colors %>% darken(0.1), guide = "none") +
  scale_y_continuous(breaks = tick_function, minor_breaks = NULL) +
  facet_grid(clade_name ~ cog_id, scales = "free", space = "free_y") +
  xlab("Species") +
  ylab("Protein abundance in orthologous groups") +
  coord_flip() +
  theme_big
```

Supplementary Figure 13: Number of proteins in each neurotransmission COGs, for every species. COGs associated with multiple neurotransmission functions are colored grey. COG0733 is associated with depletion/vesicle. KOG2301 is associated with signaling/excitability. COG0814 is associated with vesicle/synthesis.

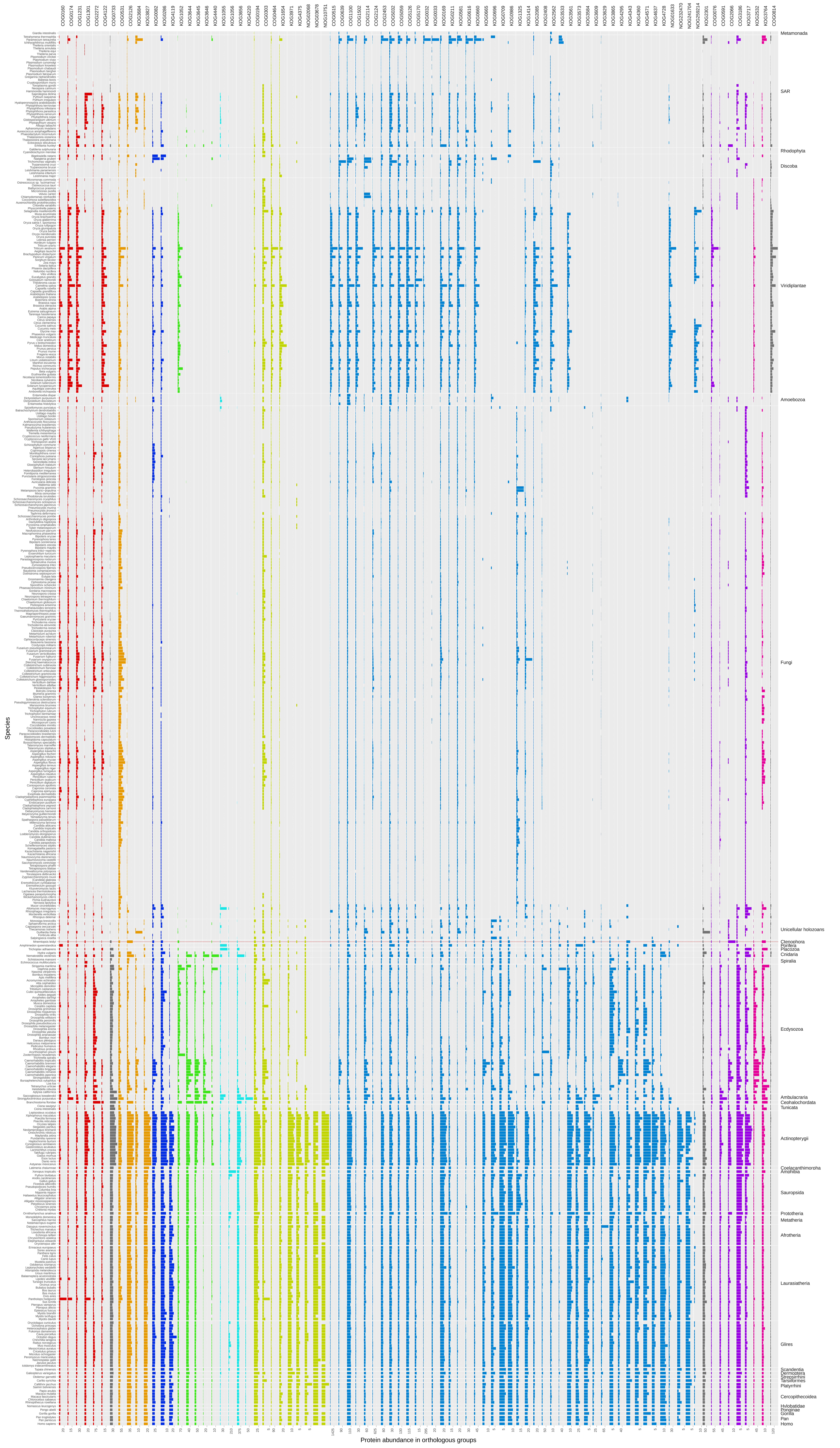

3.5 Cnidarian ionotropic receptors expression

In this section, we look for the expression of ionotropic receptor orthologs in *Nematostella vectensis* neurons, made available as supplementary data in Sebé-Pedrós et al, 2018. Loading resources:

```r
library(tidyverse)
library(magrittr)
library(readxl)
library(RCurl)
library(neurotransmissionevolution)

# Packaged data
data(
   cogs
  ,gene_ids
  ,gene_cogs
  ,gene_pathways
  ,string_eukaryotes
  ,package = "neurotransmissionevolution"
)

# Collapsing similar functions
gene_annotation <- read_tsv("../data/gene_annotation.tsv", col_types = "cc") %>%
  mutate(annotation = case_when(
     grepl("clearance",   annotation) ~ "depletion"
    ,grepl("degradation", annotation) ~ "depletion"
    ,grepl("transport",   annotation) ~ "synthesis"
    ,TRUE ~ annotation
  ))
```

Loading Supplementary Table S4, which contains genes expressed in neurons of the cnidarian *Nematostella vectensis*. The excel document contains multiple sheets, each reporting the genes expressed in a single cluster of neuronal cells. In this first step, we load all sheets into a data.frame.

```r
xlsx_filename <- "nematostella_neuronal_genes.xlsx"

download.file(
   url      = "https://ars.els-cdn.com/content/image/1-s2.0-S0092867418305968-mmc4.xlsx"
  ,destfile = xlsx_filename
  ,mode     = "wb"
)

# Loading xlsx into data.frame
nematostella_neuronal_genes <- xlsx_filename %>% excel_sheets %>% map_df(~ read_excel(xlsx_filename, .))

# We are only interested in gene names and blast best hits
nematostella_neuronal_genes %<>% select(string_name = ...1, best_hit = Hsap.BBH) %>% distinct

file.remove(xlsx_filename)
```

Next, we look for *Nematostella vectensis* genes that are associated with ionotropic receptor COGs in STRING. Then, we search for these ionotropic receptor orthologs in the supplementary table.

```r
# Gathering gene info
gene_info <- gene_ids %>%
  na.omit %>%
  left_join(gene_cogs) %>%
  left_join(gene_annotation)

# Ionotropic receptor cogs
ionotropic_cogs <- gene_info %>% filter(annotation == "ionotropic receptor") %>% pull(cog_id) %>% unique

# Nematostella taxid
nematostella_taxid <- string_eukaryotes %>% filter(string_name == "Nematostella vectensis") %>% pull(taxid)

# Finding Nematostella ionotropic receptor orthologs
nematostella_receptors <- cogs %>%
  filter(taxid == nematostella_taxid & cog_id %in% ionotropic_cogs) %>%
  select(-taxid)

# Querying STRING for gene names
nematostella_api_ids <- postForm(
   uri         ="http://string-db.org/api/tsv/get_string_ids"
```

```
  ,identifiers = nematostella_receptors %>% pull(string_id) %>% paste0(collapse="%0d")
  ,echo_query  = "1"
  ,species     = nematostella_taxid
) %>%
  read_tsv %>%
  select(string_id = queryItem, string_name = preferredName)

expressed_receptors <- nematostella_api_ids %>%
  left_join(nematostella_neuronal_genes) %>%
  left_join(nematostella_receptors) %>%
  arrange(cog_id)
```

Found receptors:

Table 18: Human ionotropic receptor orthologs expressed in Nematostella neurons, according to work done by Sebé-Pedrós et al, 2018.

COG	String ID	Gene name	Human best hit
KOG1052	EDO41291	v1g50912	Glutamate receptor 4
KOG1052	EDO41293	v1g24412	"Glutamate receptor, ionotropic, AMPA 1 variant "
KOG1052	EDO44842	v1g31895	"Glutamate receptor ionotropic, NMDA 3B "
KOG1052	EDO46596	v1g200214	"cDNA FLJ77447, highly similar to Homo sapiens glutamate receptor, ionotropic, kainate 2 (GRIK2), transcript variant 1, mRNA "
KOG1052	EDO41290	v1g104623	"Glutamate receptor, ionotrophic variant "
KOG3644	EDO30047	v1g40863	Gamma-aminobutyric acid receptor subunit beta-2
KOG3644	EDO32203	v1g134406	Gamma-aminobutyric acid A receptor beta 1 isoform A
KOG3644	EDO33939	v1g127924	Glycine receptor alpha 3 subunit
KOG3644	EDO33940	v1g127934	Gamma-aminobutyric acid receptor subunit theta
KOG3644	EDO36340	v1g11965	Gamma-aminobutyric acid receptor subunit beta-2
KOG3644	EDO36971	v1g118173	Gamma-aminobutyric acid A receptor beta 1 isoform A
KOG3644	EDO37396	v1g211643	Gamma-aminobutyric acid A receptor beta 1 isoform A
KOG3644	EDO37555	v1g230093	Gamma-aminobutyric acid A receptor beta 3 isoform A
KOG3644	EDO38214	v1g114291	"cDNA FLJ57909, highly similar to Gamma-aminobutyric-acid receptor rho-2 subunit "
KOG3644	EDO38248	v1g114264	Gamma-aminobutyric acid receptor subunit beta-2
KOG3644	EDO38572	v1g113279	Glycine receptor subunit alpha-3
KOG3644	EDO39145	v1g111552	"cDNA FLJ52443, highly similar to Glycine receptor alpha-2 chain "
KOG3644	EDO39173	v1g21524	Gamma-aminobutyric acid receptor subunit beta-2
KOG3644	EDO39193	v1g57443	GLRA1 protein
KOG3644	EDO40478	v1g22284	"cDNA FLJ52443, highly similar to Glycine receptor alpha-2 chain "
KOG3644	EDO40599	v1g207679	Glycine receptor subunit alpha-3
KOG3644	EDO40889	v1g105852	Glycine receptor subunit alpha-1
KOG3644	EDO43090	v1g204447	Gamma-aminobutyric acid receptor subunit gamma-3
KOG3644	EDO43093	v1g22641	Glycine receptor subunit beta
KOG3644	EDO43147	v1g98804	Gamma-aminobutyric acid receptor subunit beta-3
KOG3644	EDO43149	v1g98897	Gamma-aminobutyric acid receptor subunit beta-2
KOG3644	EDO43673	v1g97297	Glycine receptor subunit alpha-3
KOG3644	EDO45086	v1g93322	Glycine receptor subunit alpha-3
KOG3644	EDO45319	v1g201724	Gamma-aminobutyric acid receptor subunit alpha-5
KOG3644	EDO45677	v1g91440	Glycine receptor subunit alpha-3
KOG3644	EDO47783	v1g85497	Gamma-aminobutyric acid A receptor beta 1 isoform A
KOG3644	EDO48848	v1g82411	"cDNA FLJ57719, highly similar to Gamma-aminobutyric-acid receptor beta-1 subunit "
KOG3644	EDO49903	v1g60804	Gamma-aminobutyric acid A receptor beta 1 isoform A
KOG3645	EDO29634	v1g2281	Neuronal acetylcholine receptor subunit alpha-9
KOG3645	EDO34726	v1g214990	Neuronal acetylcholine receptor subunit alpha-9
KOG3645	EDO39536	v1g57113	Neuronal acetylcholine receptor subunit alpha-5
KOG3645	EDO43642	v1g97337	Neuronal acetylcholine receptor subunit alpha-10

KOG3645	EDO44896	v1g240779	Neuronal acetylcholine receptor subunit alpha-3
KOG3645	EDO45565	v1g91941	Neuronal acetylcholine receptor subunit alpha-3
KOG3645	EDO45734	v1g91371	Neuronal acetylcholine receptor subunit alpha-9
KOG3645	EDO47639	v1g198927	Neuronal acetylcholine receptor subunit beta-3
KOG3646	EDO42054	v1g205808	Neuronal acetylcholine receptor subunit alpha-7
KOG3646	EDO37424	v1g22673	Neuronal acetylcholine receptor subunit alpha-10
KOG3646	EDO47631	v1g85724	Neuronal acetylcholine receptor subunit alpha-7
KOG3646	EDO49735	v1g79911	Nicotinic acetylcholine alpha-7 subunit
KOG3646	EDO45735	v1g91696	Neuronal acetylcholine receptor subunit alpha-7
KOG4440	EDO35545	v1g171792	”Glutamate receptor ionotropic, NMDA 1 ”
KOG4440	EDO48986	v1g61547	”Glutamate receptor, ionotropic, N-methyl D-aspartate 1”

Unfound receptors:

Table 19: Human ionotropic receptor orthologs not found to be expressed in Nematostella neurons, according to work done by Sebé-Pedrós et al, 2018.

COG	String ID	Gene name
KOG1052	EDO27092	v1g224408
KOG1052	EDO29110	v1g4936
KOG1052	EDO41292	v1g104636
KOG1052	EDO45521	v1g36018
KOG1052	EDO46633	v1g239847
KOG1052	EDO29473	v1g221819
KOG1052	EDO29987	v1g221182
KOG1052	EDO30203	v1g141731
KOG1052	EDO32621	v1g132356
KOG1052	EDO32623	v1g13877
KOG1052	EDO32625	v1g217727
KOG1052	EDO39630	v1g110192
KOG3644	EDO25317	v1g9657
KOG3644	EDO25880	v1g9118
KOG3644	EDO25881	v1g156191
KOG3644	EDO26859	v1g224654
KOG3644	EDO27274	v1g151552
KOG3644	EDO27722	EDO27722
KOG3644	EDO28541	v1g6520
KOG3644	EDO28562	v1g147460
KOG3644	EDO29065	v1g145792
KOG3644	EDO29195	v1g145423
KOG3644	EDO29555	v1g221743
KOG3644	EDO32201	v1g134376
KOG3644	EDO34654	v1g215047
KOG3644	EDO34692	v1g125023
KOG3644	EDO35410	v1g122822
KOG3644	EDO38215	v1g52007
KOG3644	EDO38571	v1g113370
KOG3644	EDO38597	v1g113361
KOG3644	EDO40009	v1g108780
KOG3644	EDO40010	v1g12563
KOG3644	EDO40746	v1g51848
KOG3644	EDO41538	v1g103931
KOG3644	EDO42101	v1g101859
KOG3644	EDO43148	v1g99026
KOG3644	EDO43674	v1g45071

KOG3644	EDO45257	v1g92943
KOG3644	EDO45258	v1g92751
KOG3644	EDO48299	v1g198106
KOG3645	EDO27073	v1g152171
KOG3645	EDO27901	v1g149547
KOG3645	EDO30331	v1g12119
KOG3645	EDO32790	v1g247410
KOG3645	EDO33746	v1g11255
KOG3645	EDO34725	v1g124946
KOG3645	EDO34889	v1g124362
KOG3645	EDO36173	v1g120507
KOG3645	EDO37425	v1g116716
KOG3645	EDO39535	v1g51194
KOG3645	EDO39537	v1g110265
KOG3645	EDO45107	v1g202108
KOG3645	EDO45564	v1g32916
KOG3645	EDO46124	v1g200917
KOG3645	EDO46927	v1g199721
KOG3645	EDO47122	v1g87545
KOG3645	EDO49734	v1g80076
KOG3646	EDO46884	v1g87907
KOG3646	EDO47629	v1g66300
KOG3646	EDO42072	v1g60450
KOG3646	EDO26971	EDO26971
KOG3646	EDO29632	v1g46108
KOG3646	EDO30566	v1g140328
KOG3646	EDO32052	v1g40806
KOG3646	EDO32053	v1g40919
KOG3646	EDO33745	v1g128668
KOG3646	EDO36667	v1g212582
KOG3646	EDO38722	v1g112842
KOG3646	EDO38752	v1g52909
KOG3646	EDO38974	v1g50745
KOG3646	EDO39863	v1g109100
KOG3646	EDO39913	v1g109075
KOG3646	EDO40862	v1g51086
KOG3646	EDO41763	v1g103153
KOG3646	EDO42038	v1g59845
KOG3646	EDO42039	v1g205856
KOG3646	EDO47630	v1g31824
KOG3646	EDO47969	v1g85091
KOG3646	EDO48132	v1g84427
KOG3646	EDO48133	v1g198343
KOG3646	EDO49045	v1g82287
KOG3646	EDO49732	v1g79680
KOG3646	EDO42102	v1g205764
KOG4440	EDO25336	v1g226085
KOG4440	EDO27308	EDO27308
KOG4440	EDO28724	EDO28724
KOG4440	EDO30961	v1g16419
KOG4440	EDO31426	v1g219279
KOG4440	EDO32622	v1g174495
KOG4440	EDO34363	v1g11315
KOG4440	EDO35281	v1g46041
KOG4440	EDO35544	v1g15144
KOG4440	EDO37062	v1g27000

KOG4440	EDO37076	v1g212089
KOG4440	EDO37276	v1g117160
KOG4440	EDO37551	v1g211456
KOG4440	EDO47825	v1g198759
KOG4440	EDO48352	v1g83990
KOG4440	EDO48353	v1g37310

#
